# Models of service delivery in adult cochlear implantation and evaluation of outcomes: A scoping review of delivery arrangements

**DOI:** 10.1371/journal.pone.0285443

**Published:** 2023-05-10

**Authors:** Azadeh Ebrahimi-Madiseh, Mansoureh Nickbakht, Robert H. Eikelboom, Rebecca J. Bennett, Peter L. Friedland, Marcus D. Atlas, Rebecca L. Jessup

**Affiliations:** 1 UWA Medical School, The University of Western Australia, Perth, Australia; 2 Telethon Speech and Hearing, Perth, Australia; 3 Ear Science Institute Australia, Perth, Australia; 4 Centre for Hearing Research (CHEAR), School of Health and Rehabilitation Sciences, The University of Queensland, Brisbane, Australia; 5 Center for Ear Sciences, The University of Western Australia, Perth, Australia; 6 Department of Speech Language Pathology and Audiology, University of Pretoria, Pretoria, South Africa; 7 University of Notre Dame Australia, Perth, Australia; 8 School of Allied Health, Human Services and Sport, La Trobe University, Melbourne, Australia; 9 School of Medicine, Nursing and Health Sciences, Rural Health, Monash University, Melbourne, Australia; 10 Northern Health, Hospital Without Walls Service, Melbourne, Australia; University of Foggia: Universita degli Studi di Foggia, ITALY

## Abstract

**Background:**

This study aimed to describe available evidence of cochlear implantation delivery arrangements in adults and the outcomes by which these service models are measured.

**Methods:**

Scoping review of English language, primary studies conducted on adults (≥18 years) with ten or more subjects, published between January 2000 and June 2022, which assessed the effects of delivery arrangements of cochlear implantation were included. MEDLINE, EMBASE, CINAHL Plus, AMED, PsycINFO, LILACS, KoreaMed, IndMed, Cochrane CRCT, ISRCTN registry, WHO ICTRP and Web of Science were systematically searched. Included studies had to have a method section explicitly measure at least one of the Cochrane Effective Practice and Organization of Care (EPOC) outcome category. Criteria for systematic reviews and delivery arrangement category based on EPOC taxonomy was included in data extraction. Data was narratively synthesized based on EPOC categories.

**Results:**

A total of 8135 abstracts were screened after exclusion of duplicates, of these 357 studies fulfilled the inclusion criteria. Around 40% of the studies investigated how care is delivered, focusing on quality and safety systems. New care pathways to coordinate care and the use of information and communication technology were emerging areas. There was little evidence on continuity, coordination and integration of care, how the workforce is managed, where care is provided and changes in the healthcare environment. The main outcome measure for various delivery arrangements were the health status and performance in a test.

**Conclusion:**

A substantial body of evidence exists about safety and efficacy of cochlear implantation in adults, predominantly focused on surgical aspects and this area is rapidly growing. There is a lack of evidence on aspects of care delivery that may have more impact on patients’ experience such as continuity, coordination and integration of care and should be a focus of future research. This would lead to a better understanding of how patient’s view CI experience, associated costs and the value of different care models.

## Introduction

The growing demand on health systems globally has challenged providers to continuously identify and implement optimal models of care to reduce costs and improve outcomes for patients. A model of care is a delivery arrangement that aims to provide best practice for a patient population as they transition through various steps of a condition [1]. These models of care may focus on one or more aspects of how, when, or where care is delivered, who care is delivered to, or what type of care is delivered. Altering one or more of these aspects has shaped various service delivery models over the years to improve health outcomes, patient experience, sustainability and quality of healthcare services [2].

Hearing loss is a pervasive global health challenge affecting around 10% of the world’s population [3], ranking fifth on the global burden of diseases [4] with a global estimated cost of $981 billion in 2019 [5]. Age is the number one factor influencing the growing prevalence of hearing loss, and is projected to impact 900 million people by 2050 [3]. Age-related hearing loss or hearing loss as a result of an established condition may not always be curable [6], however, it can be effectively managed by hearing aids for mild to moderate degrees of loss [7], or cochlear implants (CIs) for more severe hearing loss [8].

The care cycle for adults with hearing loss involves identification and assessment (diagnosis) of hearing loss followed by intervention (auditory rehabilitation). Models of hearing healthcare (HHC) are based on the traditional and widely applied approaches to care, with referral to hearing clinics originating from primary care physicians, Ear-Nose-Throat (ENT) specialists or patients’ themselves. Hearing clinics usually require soundproof rooms and sophisticated equipment operated by HHC professionals [6, 9]. People may seek help when their hearing loss reaches a mild degree of impairment, when they may require hearing aids to manage their condition. Whether hereditary or as a result of lifestyle or environmental factors, as the hearing loss progresses [10, 11], they may require implantable devices, such as CIs, and this will involve transition from hearing aid clinics to CI units. In many countries the hearing aid based rehabilitation services are delivered by specifically trained HHC professionals (hearing aid audiologists), with a separate group of specifically trained HHC professionals being responsible for the CI eligibility assessments, CI surgery (ENT surgeons), programming and after care (CI audiologists) [12–14].

The CI models of care face a number of challenges. Firstly, relatively few potential CI candidates are referred for CI candidacy assessment [15], largely due to barriers to referral between hearing aid and CI clinics. These barriers include unclear eligibility criteria [16] and the need for continuous upskilling of the hearing aid audiologists [17, 18]. Secondly, CI services are usually centralized, offered only in specialized CI units located in metropolitan areas that provide in-clinic services [19, 20]. Centralization poses additional barriers to utilization of services for patients, particularly those from rural and remote areas. Finally, the global shortage of HHC professionals [21, 22] has made human resourcing for specialized surgical procedures and ongoing post-operative aftercare challenging; only 15% of surveyed ENT specialists in the United Kingdom identify as ear specialists [23]. The availability of an appropriately trained CI workforce is essential to the provision of high quality and effective healthcare [24]. The increasing demand for CIs from an aging population [25], the expansion of the candidacy criteria, and an improved awareness of CIs amongst both referrers and patients [26] may pose additional demands on implant units and the HHC workforce, and highlight the shortcomings of the current models of CI service delivery.

An examination of the available evidence into CI service delivery models for adult patients and how outcomes are measured may assist to identify strategies for addressing problems and improve CI services. This is the first step toward recognizing how resources might be better allocated and new models of care designed. This scoping review therefore aimed to describe the current range, extent and nature of the delivery arrangements of services provided to adult CI candidates and recipients, the extent and range of the reported outcome measures, and future research needs and priorities to improve services for this population.

## Methods

A scoping review was selected to provide a systematic method of mapping the extent and range of key concepts within the area of CI delivery arrangement research, and the nature of available evidence, given this complex and broad question has not been comprehensively reviewed before [27].

Cochrane effective practice and organization of care (EPOC) taxonomy [28] was used to categorize the delivery arrangements. The EPOC taxonomy of health system interventions provides a framework and a comprehensive inventory to systematically map the evidence about health system interventions for use in practice and policy [29, 30]. It includes four domains: delivery arrangements, financial arrangements, governance arrangements, and implementation strategies [31]. As the focus of this study was to better understand how care delivery is organised, only the domain delivery arrangements was used for classification. This domain has five categories that characterize interventions according to their conceptual, functional and/or practical similarities [29, 31]. These include (i) how and when care is delivered, (ii) where care is delivered and changes to the environment, (iii) who provides care and how the workforce is managed, (iv) co-ordination of care and management of care processes, and (v) communication and information technology, each with number of subcategories. The Cochrane EPOC also recommends a comprehensive list of primary and secondary outcome categories by which health system interventions are measured [32]. These outcome categories were used to report the outcomes in this study and are described further below.

### Protocol

The protocol was informed by the framework developed by Arksey and O’Malley [33] that was further advanced by Levac, Colquhoun [34]. This scoping review was reported according to the Preferred Reporting Items for Systematic Review and Meta-analysis for Scoping Review checklist (PRISMA-ScR) [35] (S1 File).

### Criteria for inclusion/exclusion and search strategy

The research question was formulated to be broad ensuring that delivery arrangements that fall along any point in the cycle of care for CIs, from pre-implantation to long-term post-implantation, was included. The primary question was: what types (extent, range and nature) of service delivery are available to adult cochlear implant candidates and recipients. The extent refers to the number of various delivery arrangements in each EPOC category, range refers to various types of delivery arrangements (as explained above), and nature refers to the details of methods of care delivery provided. The question was formulated in accordance with preferred reporting of scoping reviews using Population, Concept, Context (PCC) [36]. Population was adults (defined as ≥18 years old) who were CI or electric-acoustic device candidates as defined by the studies including individuals with any of the following hearing loss configurations: bilateral or in the better hearing ear having mild to profound hearing loss or having single sided deafness, pre- or post-lingual hearing loss; recipients were defined as those who have undergone CI surgery and at any stage post-surgery. Concept was service delivery, defined as delivery arrangements for CIs as an intervention, including any intervention introduced in conjunction with CIs, as well as implementation of CIs in the broader health system. This included assessment and candidacy, programming (mapping), maintenance and handling of the external device, and surgery. Delivery arrangements for cochlear implantation was formulated based on EPOC taxonomy [28]. Context was all interventions and outcomes measured pre- and post-implantation and provided in any settings. For example, urban or rural areas, face-to-face or telehealth, and public or private settings.

Included studies needed to also have explicitly measured at least one of the following outcomes according to the EPOC outcome categories:

○ Patient outcomes including health status (physical health and treatment outcome measured by mortality, morbidity and surrogate physiological outcome; psychological health and wellbeing; psychosocial outcome measured by quality of life and social activities), and health behaviors,
○ quality of care (e.g. adherence to professional guidelines),
○ access and/or utilization of healthcare services (e.g. length of stay in facilities, rate of immunization, waiting time to access services),
○ resource use (e.g. human resource/time, consumables, transport, informal care giver time),
○ impacts on equity and/ or social outcomes (e.g. community empowerment, poverty measures, employment, education),
○ healthcare provider outcomes (e.g. workload, work morale, stress, burnout, absenteeism),
○ adverse effects (e.g. balance disturbance),
○ knowledge, attitude, satisfaction and performance in a test situation as secondary outcomes.

Studies were excluded if: they reported a mix of adults and pediatrics (except those separately reporting results for adults and pediatrics participants); were in a language other than English; conducted before 2000; conducted on cadaver, temporal bone or animals; reported developments of CIs technology and tools; described experimental phases of devices and electrodes, coding strategies, programming and changes in parameters or in simulated conditions; or were solely focused on effectiveness or efficacy of CIs, hearing preservation, comparison of electrodes and surgical techniques. Qualitative studies, case studies (<10 subjects), editorials and review reports, grey literature, doctoral theses, opinions, and conference proceedings were also excluded. With the reviews and systematic reviews, a snowballing approach was implemented to include referenced studies that met the inclusion criteria. No restriction was put on the type of study/trial to ensure the breadth of the topic was captured.

A wide range of databases and resources were searched. The search strategy was systematic but also iterative to ensure the breadth of existing evidence was captured. Publications between 1^st^ January 2000 to June 30^th^ 2022 were considered. The search strategy is described in S2 File. The following databases were searched: MEDLINE (Ovid), EMBASE, CINAHL Plus (EBSCO), Allied and Complementary Medicine Database (AMED), PsycINFO, LILACS, KoreaMed, IndMed, Cochrane Central Registered of Control Trials (CENTRAL), ISRCTN registry, WHO ICTRP and Web of Science. These databases capturing publications in hearing healthcare and healthcare management. They were all included to ensure the breadth of the existing literature was captured. The search results for each database were exported to EndNote to remove duplicates and then to Rayyan systematic review assistance application [37] for assessment of eligibility for inclusion.

### Selection of studies

**Fig 1:**
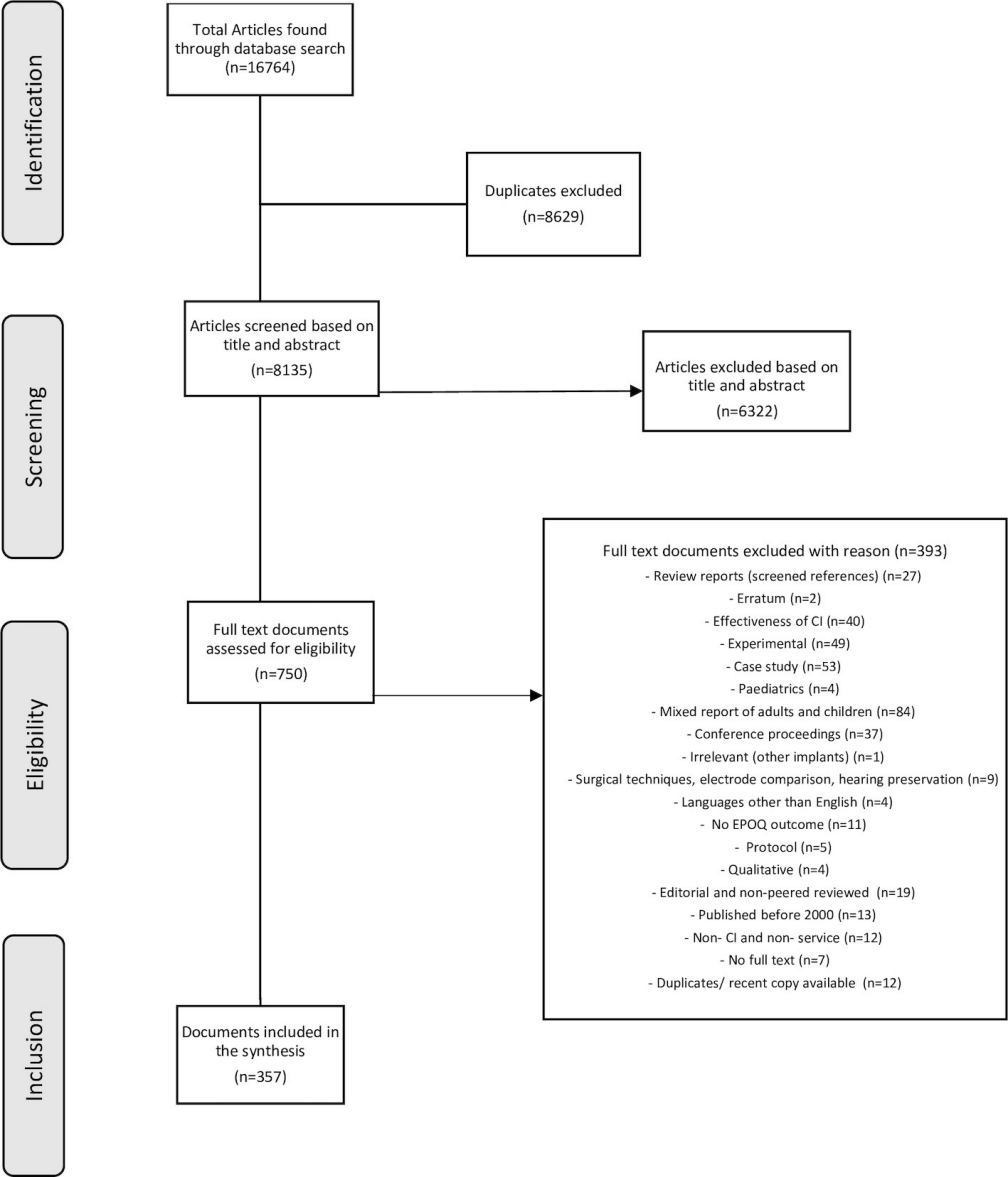
PRISMA flowchart describing the process and number of excluded/included studies.

Three authors (AEM, RHE and RJB) independently screened the titles and abstracts of 20 randomly selected articles to test the inter-rater reliability of the application of inclusion and exclusion criteria described above. Reasons for inclusion and exclusion were recorded to evaluate the agreement between the independently reviewed articles. As there was a high agreement between the independent screenings (97%), the rest of the studies were screened by the primary author (AEM). Where a decision could not be made based on the title and abstract, the full text was reviewed. Screening of the full text was conducted by two authors (AEM, MN). Fig 1 shows the PRISMA flowchart summarizing the study selection process.

### Data extraction and management

Full texts of all included primary studies were reviewed to extract data related to delivery arrangement strategy and outcome. Two authors (AEM and MN) extracted the following data: study characteristics (author, year of publication, journal, type of study), place published, objective of the study and study design, brief description of findings and /or intervention, population and sample, outcome type (based on Cochrane EPOC outcome category), delivery arrangement strategy (based on Cochrane EPOC taxonomy). Data were tabulated in Microsoft Excel and crosschecked between the two extracting authors to reach agreement on the outcome type and delivery arrangement categorized under each sub-category. An additional author (RHE and RJB or PF) were consulted if disagreements could not be settled until consensus was reached on the delivery arrangement and EPOC outcome categories. The process was overseen, and decisions confirmed by the senior author (RLJ).

### Collating and summarizing the data

Delivery arrangements were categorized and summarized using the five categories of Cochrane EPOC taxonomy of health system interventions as previously described. The authors added a sixth category for studies with ‘specific goals’ which focused on current practice or on an economic analysis of CI. Studies were categorized based on their aims. In the instance where a study investigated two or more of the EPOC categories, it was categorized according to the primary aim of the study. The findings were numerically reported as the number of studies in each tabulated item and visually presented with charts to display the quantity and range of studies in each category informed by Jessup et al. [38, 39] on alternative models of service delivery. The data was also narratively synthesized to describe the findings relative to the aim of the study and implications for future research. The level of evidence of the included studies was categorized according to the Australian National Health and Medical Research Council (NHMRC) hierarchy of evidence [40].

## Results

### Search results

The literature search generated 16,764 records. After removal of duplicates (n = 8629), titles and abstracts of records (n = 8135) were screened for inclusion. Full text records (n = 750) were retrieved and further assessed for eligibility. Following exclusion of 393 full text papers, the remaining 357 papers were included in the data extraction and synthesis phases (Fig 1).

### Description of included papers and outcome measurements

**Fig 2:**
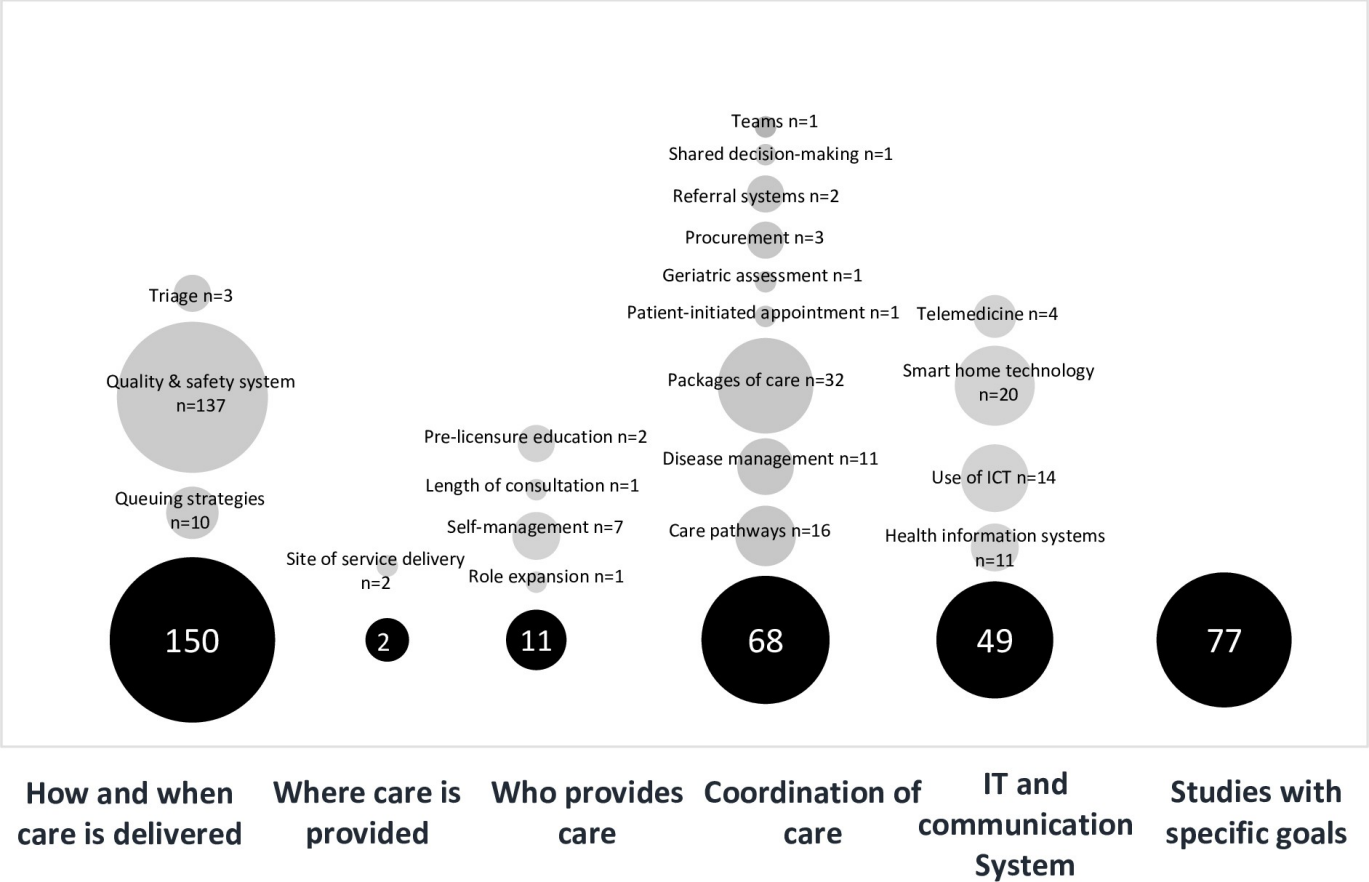
Number of included studies in synthesis organised according to the EPOC taxonomy of delivery arrangements and a seventh category for studies with specific goals. The size of circles illustrates the number of studies in each category and subcategory.

More than 75% of the included studies were published after 2010. Studies were conducted across a range of countries with the largest representation being from the United States of America (36.7%), followed by the United Kingdom (11.4%), Germany (11.3%) and Australia (6.3%). The remaining 34.3% originated from 27 countries, each contributing <5% to the total number of included studies. Observational study designs were used for 90% of the studies with the level of evidence being ranked between III-2 and IV. Interventional studies accounted for 30.3% of the studies of which 3% were randomized controlled trials, classified between II and III-3 for the hierarchy of evidence. Fig 2 provides an overview of the included studies in each EPOC delivery arrangement category, descriptively described further below. The citation of included papers is described in S3 File.

**Fig 3:**
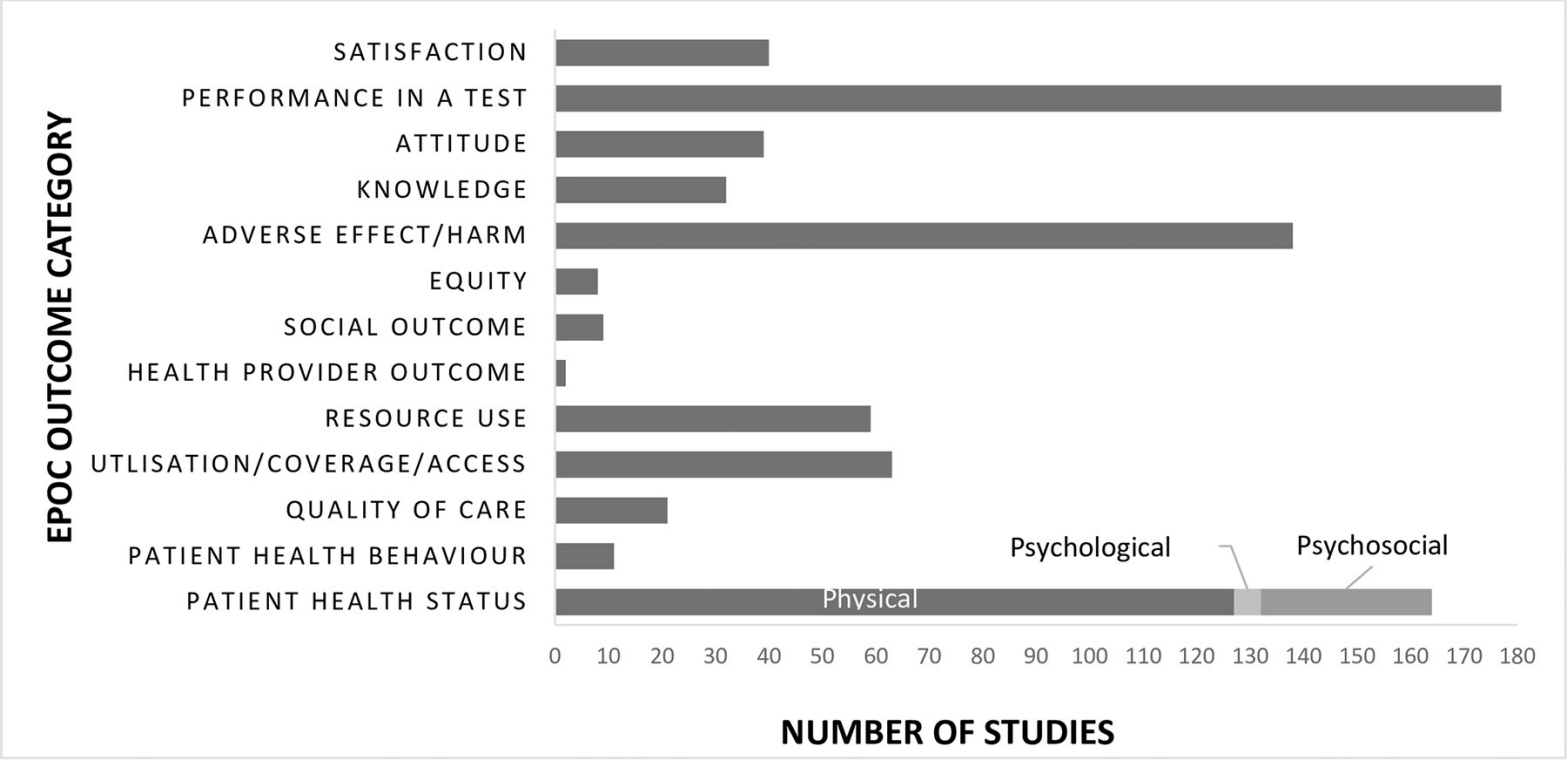
Nature and number of outcome measures in the included studies according to EPOC outcome category.

Included studies measured a range of primary and secondary outcomes, with patient outcomes, performance in a test situation, and adverse effect/harm dominating the remainder (Fig 3). Studies that investigated how care is delivered focused on quality and safety of current CIs and interventions to improve the quality and safety of the CI procedure. Outcome measures in the majority of these studies (n = 114) were patient health status (morbidity and physiological measures) followed by adverse effects or harm (n = 110). Coordination of care included studies with a focus on performance in a test as the main outcome measure (predominantly speech perception tests) followed by patient outcomes of health status, physical health and patient reported outcome measures (PROMs) such as quality of life (QoL). Similarly, studies that evaluated the use of information and communication technology to provide and/or improve services chose performance in a test as their main outcome measure, followed by satisfaction in a test condition and patient outcomes of health status (physiological measures and PROMs). Main outcome measures in studies that investigated who provides care were performance and satisfaction in a test situation, with two also measuring health behavior (adherence to the interventions). Resource use, service utilization and satisfaction were the main outcome measures in studies that examined when care is provided. Only two studies looked at where care is provided in delivering services with the main outcome measure being performance in a test. Studies that were categorized in the ‘specific goals’ category measured a range of primary and secondary outcome measures including utilization, access and coverage, resource use, quality of care and secondary outcomes of knowledge, attitude and performance in a test.

### How and when care is provided

These studies described essential standards of safety and quality of surgical aspects of implantation including the safety profile of Cis, and interventions to reduce and/or manage peri- and postoperative morbidities and poor outcomes. Studies that investigated the safety profile of CIs (n = 63) described the profile and timing of various minor and major complications peri- and post operation in younger and older adults, MRI and anesthetics safety, rate of revisits, and safety profile in patients with specific co-morbidities. The remainder of the studies describe interventions to improve quality of surgical outcomes, for example, immunization guidelines, CO_2_ laser assisted or under-water surgery to improve accuracy of electrode insertion and usage of various imaging techniques to ascertain electrode positioning peri and post operation. The 14 studies that focused on when care is provided to CI patients investigated how timing of the care provided impacts the outcome and/ or patients including changes in time to access CIs, factors to optimize operation time, proposed models for same day CI consultation and implantation, early activation of speech processors and optimizing client triaging system. One study looked at the impact of COVID-19 on CI surgeries. Details of these studies and the number of studies in each subcategory is summarized in Table 1 and additional file 3 (citations).

**Table 1:** Summary of the included studies organized according to the Cochrane EPOC taxonomy of delivery arrangement.

**Delivery arrangement category**				
**How and When care is delivered (n = 150)**				
*Sub-category*	*Definition*	*Number of studies (n) (observational studies*, *n)*	*Non-RCT (n) (RCT*, *n)*	*Details–Citation and details of all studies provided in supplementary file 3*.
**Group versus individual care**	Comparisons of providing care to groups versus individual patients, for example intensive group therapy, group vs. individual antenatal care.	0	-	
**Queuing strategies**	A reduction or increase in time to access a healthcare intervention, for example managed waiting lists, managing ER wait time.	10 (9)	1 (0)	○ Surgical duration for CIs and influencing factors (unilateral, bilateral, revision and re-implantation, obesity) ○ Same day cochlear implant consultation and implantation; patient satisfaction with the model ○ Early activation of CIs ○ Impact of surgical waiting time on psychosocial wellbeing of CI candidates ○ Impact of inter-implant intervals ○ Improving time to access services pre and post op
**Coordination of care amongst different provider**	Organizing different providers and services to ensure timely and efficient delivery of healthcare.	0	-	
**Quality and safety systems**	Essential standards for quality of healthcare, and reduction of poor outcomes related to unsafe healthcare.	137 (132)	2 (3)	○ Safety profile of CIs (post-op infection, vestibular and balance disturbance, device failure, taste disturbance, anaesthetics, CI in specific cases) ○ MRI safety in cochlear implant recipients ○ Interventions to quality control intra-cochlear electrode positioning peri and post-op (X-Ray, rotational tomography, co-registered cone beam CT scan and MRI, cone bean CT scan, flat panel CT scan, ECochG and CT scan, fluoroscopy) ○ Interventions to reduce post cochlear implant morbidities (antibiotics, skin flap and magnet displacement management, minimal hair shave, facial nerve palsy, pain management and opioids) ○ Adherence to pre-op immunisation guidelines ○ Interventions to improve surgical outcomes (application of steroids, under water surgical techniques, Co2 laser assisted surgery) ○ Diagnostic utility of pre-op imaging in surgical management decision-making ○ Surgical approach to improve safety and efficiency ○ Interventions to evaluate and manage vestibular damage peri and post cochlear implantation (utility of vHIT, vestibular rehabilitation, application of VEMP) ○ Revision CI surgery and re-implantation to manage complications ○ Interventions to manage non-auditory stimulation ○ Interventions to manage hard-failure ○ Interventions to diagnose, reduce or improve electrode migration
**Triage**	Management of patients attending a healthcare facility, or contacting a healthcare professional by phone, and receiving advice or being referral to an appropriate service.	2	0 (0)	○ Improving patient flow (Same day triage system model) and patients’ satisfaction with the model
**Where care is provided and changes to the healthcare environment (n = 2)**				
*Sub-category*	*Definition*	*Number of studies (n) (observational studies*, *n)*	*Non-RCT (n) (RCT*, *n)*	*Details*
**Environment**	Changes to the physical or sensory healthcare environment, by adding or altering equipment or layout, providing music, art.	0	-	
**Outreach services**	Visits by health workers to different locations, for example involving specialists, generalists, mobile units.	0	-	
**Site of service delivery**	Changes in where care is provided, for example home vs. healthcare facility, inpatient vs. outpatient, specialized vs. non-specialized facility, walk in clinics, medical day hospital, mobile units.	2 (2)	0 (0)	○ CI programming and candidacy evaluation at private and community settings
**Size of organization**	Increasing or decreasing the size of health service provider units.	0	-	
**Transportation services**	Arrangements for transporting patients from one site to another.	0	-	
**Who provides care and how the healthcare workforce is managed (n = 11)**				
*Sub-category*	*Definition*	*Number of studies (n) (observational design*, *n)*	*Non-RCT (n) (RCT*, *n)*	*Details*
**Role expansion of task shifting**	Expanding tasks undertaken by a cadre of health workers or shifting tasks from one cadre to another, to include tasks not previously part of their scope of practice.	1 (1)	0 (0)	○ Psychosocial counseling skills for audiologists
**Self-management**	Shifting or promoting the responsibility for healthcare or disease management to the patient and/or their family.	7 (6)	0 (1)	○ Self- assessment and home-based evaluation of post-operative progress in CI recipients ○ Self-programming of CI external processors ○ Self-help cognitive behavioral therapy program
**Length of consultation**	Changes in the length of consultations.	1 (1)	0 (0)	○ Faster map generation in an appointment
**Staffing models**	Interventions to achieve an appropriate level and mix of staff, recruitment and retention of staff, and transitioning of healthcare workers from one environment to another, for example interventions to increase the proportion of healthcare workers in underserved areas.	0	-	
**Exit interviews**	A verbal exchange or written questionnaire between employees’ resignation and last working day.	0	-	
**Movement of health workers between public and private care**	Strategies for managing the movement of health workers between public and private organizations.	0	-	
**Pre-licensure education**	Changes in pre-licensure education of health professionals.	2 (1)	1 (0)	○ Postgraduate specialization fellowship for audiologists ○ Intervention to improve counseling skills (Narrative competence)
**Recruitment and retention strategies for underserved areas**	Strategies for recruiting and retaining health workers in underserved areas.	0	-	
**Recruitment and retention strategies for district health managers—LMIC**	Interventions for hiring, retaining and training district health systems managers in LMIC.	0	-	
**Coordination of care and management of care processes (n = 68)**				
*Sub-category*	*Definition*	*Number of studies (n) (observational design*, *n)*	*Non-RCT (n) (RCT*, *n)*	*Details*
**Care pathway**	Aim to link evidence to practice for specific health conditions and local arrangements for delivering care.	16 (14)	(2)	○ Remote follow up pathway for cochlear implant recipients ○ Clinical care pathway for patients with SSD ○ Anesthetics care pathway for cochlear implantation; local vs general (Safety, cost, effectiveness, patient satisfaction) ○ Evidence-based cochlear implant selection criteria ○ Comprehensive self- administered CI selection test
**Case management**	Introduction, modification or removal of strategies to improve the coordination and continuity of delivery of services i.e. improving the management of one “case” (patient) or one individual to provide care.	0	-	
**Communication between providers**	Systems or strategies for improving the communication between health care providers, for example systems to improve immunization coverage in LMIC.	0	-	
**Comprehensive geriatric assessment**	A multidimensional interdisciplinary diagnostic process focused on determining a frail older person’s medical, psychological and functional capability to ensure that problems are identified, quantified and managed appropriately.	1 (1)	-	○ Improving assessment of elderly in otolaryngology clinics, physical performance battery
**Continuity of care**	Interventions to reduce fragmented care and undesirable consequences of fragmented care, for example by ensuring the responsibility of care is passed from one facility to another so the patient perceives their needs and circumstances are known to the provider.	0	-	
**Discharge planning**	An individualized plan of discharge to facilitate the transfer of a patient from hospital to a post-discharge setting.	0	-	
**Disease management**	Programs designed to manage or prevent a chronic condition using a systematic approach to care and potentially employing multiple ways of influencing patients, providers or the process of care.	11 (11)	-	○ Hearing management in patients with head trauma ○ Management of patients with NF2 with CI: decision making tool for CI vs ABI; CI without tumour removal; comparison of CI outcome with and without tumour removal; Comparison of CI outcome in irradiated and non-irradiated ears; CI in unilateral vestibular Schwannoma
**Integration**	Consolidating the provision of different healthcare services to one (or simply fewer) facilities.	0	-	
**Packages of care**	Introduction, modification, or removal of packages of services designed to be implemented together for a particular diagnosis/disease, e.g. tuberculosis management guidelines, newborn care protocols.	32 (29)	(3)	○ Alternative test materials for testing patients with CI: AB words test as a candidacy test; non-linguistic tests for candidacy; non-linguistic tests to follow up progress; using TEN test for CI eligibility ○ Use of objective measures to assist CI fitting: use of aided CAEP in SSD CI users; image-guided maps in CI users (IGCIP), image-based electrode deactivation reprogramming technique (IBEDRT) ○ Use of auditory and communication training packages to improve outcome post-CI: use of intensive psychophysical auditory training; auditory verbal skill training (AVST); combination of speech and sign therapy (Sim-Com) for improving communication in noise; communication strategy therapy in older adults; Digit in noise training; modulated telephone signal for telephone rehabilitation therapy; structured group-based therapy communication program; Phoneme training in older adults ○ Music therapy program: Individual, face-to face; web-based instrument recognition therapy; gamification of therapy ○ Use of objective measures to improve decision making in cochlear implantation: prognostic value of fNIRS for CI outcome prognostic value of radiodensity in measurement of cochlear ossification and fibrosis; scoring system to predict CI suitability in Sporadic VS; imaging for pre-op skin flap measurement ○ Robot-assisted electrode insertion ○ Utility of ultrasound in diagnosis of magnet dislocation
**Patient-initiated appointment system**	Systems that enable patients to make urgent appointments when they feel they cannot manage their condition or where something has changed unexpectedly.	1 (1)	-	○ Traditional vs. patient-led postoperative review appointments
**Procurement and distribution of supplies**	Systems for procuring and distributing drugs or other supplies.	3 (3)	-	○ Impact of financial incentives in cochlear implant access ○ Impact of Medicaid on cochlear implant access (USA) ○ Impact of surgical mark up on cochlear implantation (USA)
**Referral system**	Systems for managing referrals of patients between health care providers	2 (2)	-	○ Cochlear implant referrals from hearing aid to cochlear implant clinics ○ Intervention to improve cochlear implant referrals from hearing aid audiologists
**Shared care**	Continuing collaborative clinical care between primary and specialist care physicians.	0	-	
**Shared decision making**	Sharing healthcare decision making responsibilities among different individuals, potentially including the patient.	1 (1)	-	○ Agreement of cochlear implantation success between cochlear implant recipients and significant others
**Teams**	Creating and delivering care through a multidisciplinary team of healthcare workers.	1 (1)	-	○ Shared Medical Appointment to improve patient flow
**Transition of care**	Interventions to improve transition from one care provider to another, for example adolescents moving from child to adult health services.	0	-	
**Information and communication technology (ICT) (n = 49) **				
*Sub-category*	*Definition*	*Number of studies (n) (observational studies*, *n)*	*Non-RCT (n) (RCT*, *n)*	*Details*
**Health information system**	Health record and health management systems to store and manage patient health information, for example electronic patient records, or systems for recalling patients for follow-up or prevention e.g., immunization.	11 (11)	-	○ Long term follow up of CIs through a national and international database: Function, device use and complications; Adverse events ○ Digitisation of the ENT health records for CI patients ○ Digital multi-faceted protocol to improve pneumococcal vaccination rate in hospitals ○ Use of a national CI registry to determine CI candidacy
**The use of information and communication technology**	Technology based methods to transfer healthcare information and support the delivery of care.	14 (13)	(1)	○ Use of VR in training (ENT registrars trained for CI surgery) ○ A tablet-based tool to assist surgeons in electrode insertion ○ Digital awareness campaign for CI in older adults ○ Modelling data and data mining: screening tool to identify CI candidates; screening tool to identify second side CI candidates ○ Image guided mapping at a distant site ○ Machine learning and automated changes in maps: Using FOX2 software; FOX software; Machine learning and postoperative outcome prediction ○ Multimedia digital support tool to educate potential CI candidates ○ Web-based information for consumers about CI
**Smart home technologies**	Electronic assistive technologies.	20 (20)	-	○ Web-base at home auditory training packages: phonemes and words; music training (The Hear Tunes software) ○ Wireless home technologies: phone clip to improve understanding on phones by CI users, bimodal users; CROS MIC to improve speech in noise understanding and localisation in unilateral CI users, in bilateral users; remote MIC to enhance speech understanding in noise in bimodal users; use of Roger FM system in SSD CI users ○ Smart phone application for tinnitus relief in CI users; assess progress with CI post implantation
**Telemedicine**	Exchange of healthcare information from one site to another via electronic communication.	4 (4)	-	○ Remote programming of CIs external processor ○ Telemedicine for postoperative care
**Studies / interventions with specific goals (n = 77)**				
*Sub-category*	*Definition*	*Number of studies (n) (observational studies*, *n)*	*Non-RCT (n) (RCT*, *n)*	*Details*
**Current practices in hearing healthcare to manage potential and existing CI recipients (Candidacy and referral, fitting, surgical choice).**		19 (19)	-	○ Current practices and attitude in CI programming in audiology clinics: bimodal fitting; mapping of the external processor ○ Current practices and knowledge and attitude of CI audiological and surgical candidacy assessment: in ENT surgeons; ENT surgeons providing CI services to humanitarian programs; non-ENT surgeons; audiologists; second side CI candidacy; international differences in candidacy and recommendations ○ Current practices of primary care physicians in CI referrals ○ Knowledge and current practice of vocational Rehabilitation Counsellors about CI ○ Current service provision to older adult CI candidates and recipients
**Economic analysis of CI services: cost analysis of CIs, auditory training and pre-op imaging; cost utility analysis; cost effectiveness analysis of CIs, and impact of CI on the income and employment of recipients.**		20 (10)	9 (1)	○ Cost- ***effectiveness*** of CI: unilateral CI in public setting; impact of age on cost effectiveness of CIs compared to hearing aids in high income countries ○ Cost analysis of CI: surgical and first year rehabilitation cost of CI in France; sequential vs. simultaneous CIs in USA; life time cost of unilateral CI in adults in Germany; pre-operative imaging cost in post-lingual adults; cost analysis of various modes of auditory training ○ Cost utility analysis of CIs: bilateral CIs, long term costs of bilateral CIs in publicly funded setting, simultaneous bilateral from insurance perspective; unilateral CI ○ Personal economics and societal benefit of CI for recipients; Employment and employment retention in CI recipients
**Population or individual-based epidemiological studies: prevalence of CI in adults, hearing and socioeconomics characteristics of CI candidates and recipients, rates of CI uptake, device use and utilisation of healthcare.**		33 (33)	-	○ Prevalence of CI in adult: prevalence of CIs in postlingually deafened adults and severe to profound HL in Sweden; prevalence of CI in Europe; prevalence of CI and EAS in Japan; prevalence of CI in elderly in public system in USA; Prevalence and characteristics of hearing management in the USA; prevalence and growth in USA ○ Hearing and socioeconomic profile of CI adult candidates and recipients: hearing profile in the USA; hearing profile and service trends in Canada; socioeconomics and equality profile comparison between urban and rural areas in the USA; in SSD; In second side CI ○ Device use in CI candidates and recipients: rate of hearing aid use in the non-implanted ear and influencing factors; rate of hearing aid use in CI candidates and correlation with the uptake of CI; rate and cause of elective CI non-use amongst CI recipients; CI use and satisfaction ○ Rate of CI uptake: rate and correlation with demographic and socioeconomic factors; rate of uptake and patients’ perspective for non-adoption, audiometric configuration and uptake; racial disparity; CI profile and catchment (USA) ○ Rates of healthcare utilisation and subsequent management in elderly post-CI: short term post-CI compared to younger adults; long term audiological service utilisation and management
Patients’ awareness and attitude about hearing and tinnitus management. Care givers of CI recipients		4 (4) 1 (1)	- -	○ Awareness of and attitude towards HL management in older adults; Attitude and acceptance of invasive treatments for tinnitus amongst patients; Knowledge and attitude about MRI; Public attitude and knowledge about CI ○ Quality of life of care givers of CI recipients after cochlear implantation

### Where care is provided and changes to the healthcare environment

Only three studies were identified for this category. Two studies investigated where care is provided by comparing the postoperative mapping of a decentralized network of non-implant audiology clinics with the routine mapping in the implant clinics. The other study looked at the utility of pure tone audiometry conducted at community clinics to ascertain CI candidacy.

### Who provides care and how the healthcare workforce is managed

The eleven studies identified in this category included self-management of postoperative care processes, interventions to reduce the length of postoperative audiological consultation, role expansion, and pre-licensure training for audiologists. The self-management studies focused on self-assessment of postoperative progress and fine-tuning of the external device by recipients. Up-skilling audiologists and students to better address psychosocial consequences of hearing loss, improving therapeutic relationships, and general attitude and acceptance toward these approaches was also explored (Table 1)

### Coordination of care and management of care process

The majority of the 68 studies in this category were in three subcategories: care pathways, disease management, and packages of care. These studies investigated new care pathways for CIs, management of specific chronic conditions with CIs, introduction or modification of care packages for CI recipients and candidates, referral and patient-initiated appointment systems, impact of procurement on access to CIs, shared decision making between CI recipients and families, and a multidisciplinary approach to CI care delivery.

In the care pathway subcategory, studies specifically explored a range of surgical and audiological pathways to link evidence to practice. The application of a remote follow-up system for CI recipients to reduce the number of face-to face appointments, clinical care decision making for patients with single sided deafness (SSD), application of local anesthetics in CIs to reduce cost and increase safety of the surgical procedure, use of photography to facilitate decision making of complications and skin management, and providing evidence-based selection criteria for CIs were included in this sub-category.

Within the disease management subcategory, cochlear implantation was also investigated as an intervention to manage special cases compared with other interventions and at various stages of the diseases.

The packages of care subcategory contained the largest number of studies and incorporated tests, tools, treatments and programs that intend to supplement and/ or improve the routine care delivery to CI candidates and recipients. These covered both audiological and surgical aspects of cochlear implantation, including alternative speech tests to assess CI candidacy and progress, objective tests to guide programming of the external device, imaging to improve the prognostic measures for cochlea ossification and fibrosis and to better predict CI outcomes and magnet dislocation, robot assisted electrode insertion, and a range of auditory, communication and music therapy.

### Information and communication technology (ICT)

The majority of studies in this category focused on smart home technologies, specifically examining technologies that have been or could be utilized at home by CI recipients to enhance their outcome and performance in various situations. The use of ICT to support the delivery of care, address lack of resources, and to educate potential candidates about CIs were investigated in 14 studies. Also included were health information systems that examined the use of, and improvements to, patient data management systems, the use of big data to inform better decision-making, and the exchange of healthcare information via electronic communication such as remote programming of the CIs and predicting CI candidacy.

### Studies with a specific focus

Studies included in this group did not directly look at any categories of delivery arrangements based on EPOC categories. The aim of these studies was indirectly related to some aspects of service delivery and met the inclusion criteria, hence included in this scoping review. These studies evaluated knowledge, attitude and current practices of a range of professionals about various aspects of service provision to CI patients including cost effectiveness and utility analysis of CIs and postoperative interventions (e.g. auditory training), epidemiological or single-center investigations on prevalence and growth of cochlear implantation and socioeconomic profiles, CIs utilization, uptake and associated factors in various countries.

## Discussion

This study aimed to describe the range, extent, and nature of the delivery arrangements of service models provided to adult CI patients and the range of outcomes by which these services are measured. This is the first scoping study of its kind which examines the evidence for various service delivery models in adult cochlear implantation. Such a broad overview can help hearing healthcare providers, policymakers, and other stakeholders to identify strategies for addressing problems and ultimately improving the overall health systems.

We identified that most of the studies investigating how care is provided in adult cochlear implantation focused on quality and safety systems. The insertion of an electrode in a delicate structure like the cochlea demands careful investigation and testing to ensure high quality outcomes [41], and to this end approximately 40% of all the published studies captured by this review have focussed on this. However, the preoccupation over the past 22 years with the surgical aspects and/or associated morbidities of cochlear implantation has directed research attention away from other aspects of service delivery that may be of equal importance in determining good patient outcomes such as factors that influence patient access to, and engagement with, care. In comparison, a recent scoping review of systematic reviews on alternative models of healthcare service delivery in all disciplines, found only 8% of primary studies focused on this EPOC category [38].

A significant finding in this study is the paucity of evidence on where care in provided to adult CI recipients and candidates, as well as potential changes to the healthcare environment that might improve service delivery. Location and availability of healthcare services is an important determinant of access to services and equity in healthcare [42]. Centralization of specialist health care services may allow for greater volume and efficiencies, facilitation of training, and reduction in costs and clinical variability [43, 44]. However, it also requires patients to have the ability and willingness to transition from their usual HHC provider (hearing aid audiologist) to travel to major centers. Provision of telehealth for some aspect of CI service delivery may provide opportunities to improve access and equity by reducing the burden of travel [45, 46].

The increasing use of digital health provides a promising means to improve access, equity, quality and sustainability in healthcare [47, 48]. However, the use of ICT in service delivery accounted for only a small number of studies in this review, with the majority focusing on assistive technologies to improve speech in noise understanding. However, there was an emerging field of research using data in machine learning and artificial intelligence to improve outcome prediction [49], post-operative programming of external devices [50], and improved interoperability of patients’ data using national health records [51]. These strategies, combined with self-management solutions for postoperative care for CIs [52], might also address HHC workforce shortages [53, 54].

A limited number of studies in this review investigated how the HHC workforce is managed, and how teams are coordinated to provide services to the whole cycle of care for cochlear implantation. This gap in knowledge provides an opportunity for further research, specifically in designing integrated models of care where services are organized around the health condition and needs of the patient. These models have shown to improve service utilization and patient experience [55], reduce the cost of care [56] and create better value for the healthcare system [57].

Lastly, most studies across categories focused on patient health status outcomes only, specifically with a narrow focus on surgical survival and morbidity. Similar findings were reported in a study evaluating the range and extent of outcome measures for auditory rehabilitation with hearing aids [58] where patient health status outcomes were the main outcomes measured although in the areas of psychological and health behaviour outcomes. Unsurprisingly, the second most frequently measured outcome in this study was performance in a test situation, usually measured by speech perception tests, with 33% of studies reporting this as their only outcome measure. These measures of speech perception have historically determined the effectiveness of CIs [8] and are the basis of the minimum surgical [59] and audiological [60] outcome reporting requirements. While there is an acknowledged consensus on what core outcomes should be measured and reported in hearing healthcare [61], these outcome measures are episodic, organized around the intervention [62] and/ or provider [63], and hence fall short of measuring the outcome for the whole cycle of care delivery especially the experience of care delivery. While outcome measures like mortality and morbidity will remain useful, they provide minimal information about the interaction of patients with the healthcare system and delivery of care. In the past years, the urgency of putting people at the center of the healthcare systems has gained momentum [63]. In a recent report by the Organization of Economic Cooperation and Development (OECD) measuring the experience of patients using Patient Reported Outcome Measures (PROMs) and Patient Reported Experience Measures (PREMs) is paramount to not only better understand how healthcare delivery and health policy affect the lives of people, but to help inform decisions and ensure the delivery of high value care. Future research should focus on the PROMs and PREMs associated with the provision of CIs, as a positive patient experience is associated with improved clinical outcomes [64, 65].

Furthermore, to evaluate the value of a model of care, measuring the cost and reporting the economic evaluation of interventions is essential. This scoping review highlighted that outcomes such as resource use, utilization and coverage, or social outcomes were rarely measured in CI service delivery literature. Only 4% of the studies evaluated the economic impact of CIs or other interventions for CI patients. These outcomes may be of particular interest to decision makers in healthcare management and policy makers to appropriately (re)allocate the finite resources.

The findings of this review have implications for future research. The shortcomings of current outcome measures raise the question of how services are evaluated from the patient’ perspective and what factors influence the lived experience of patients interacting with the healthcare system. If the value of a given intervention or service model is measured by the outcome per dollar spent [66], measuring the right outcome is crucial. The advent of digital health for use in hearing healthcare provides significant opportunities to create new models of care allowing for personalization of care in a range of outcomes that matter to patients, improved access and equity, improved efficiency of care, as well as potentially addressing workforce issues, all of which have yet to be investigated adequately for CI service delivery.

There are several strengths and limitations to this scoping review. This study is the first to use Cochrane EPOC to evaluate the delivery arrangements of CI service models. In addition, a robust method for assessing studies for inclusion was used, with three authors independently having screened and selected reviews with a high inter-rater agreement between the inclusion/ exclusion, thus minimizing the likelihood of excluding eligible reviews. While independent data extraction was conducted by two review authors, we took steps to optimize data extraction consistency by crosschecking in the process. As this scoping review sought to map the literature in this area, a limitation is that we did not appraise the quality of the methodology or reporting of the included studies. Additionally, while we used the Cochrane EPOC taxonomy for delivery arrangements to map the extent, range and nature of the evidence in the studies, categorization was not always straightforward and different authors may have categorized the studies differently. This was because interventions could sometimes be categorized into more than one category. In these cases, the aim of the study and crosschecking guided the decision.

## Conclusion

A substantial body of evidence exists about the safety and efficacy of cochlear implantation in adults, and this is predominantly focused on surgical aspects. The application of digital health in improving hearing healthcare services in a growing body of literature and further investigation might demonstrate how ICT might reduce inequity and access issues in delivery of CI care. There was a paucity of evidence on continuity, coordination, and integration of care, how the workforce is managed, where care is provided, and changes in the healthcare environment and these delivery arrangement areas may warrant focus in future research. In addition, better understanding about how patients and broader stakeholders view CI experience and associated costs across the whole cycle of care delivery is required. Addressing knowledge gaps relating to broad and holistic clinical and patient experience outcome measures and cost analysis of interventions and/or services would provide a better understanding of the value of care models based on what matters most to the patients.

## Supporting information

S1 File Preferred reporting items for systematic reviews and meta-analyses extension for scoping reviews (PRISMA-scr) checklist. (PDF)

S2 File Sample search strategy. (PDF)

S3 File List of citations in each EPOC delivery arrangement category. (PDF)
