# Supplementary material for: Models of service delivery in adult cochlear implantation and evaluation of outcomes: A scoping review of delivery arrangements
Source: PLoS One. 2023 May 10;18(5):e0285443. doi: 10.1371/journal.pone.0285443 (PMC10171603; doi:10.1371/journal.pone.0285443)
Supplement: S2 File — (PDF) [file pone.0285443.s002.pdf]

Supplementary File 2; Sample search strategy

Database: Ovid MEDLINE(R) January 2000 to June 2022
Advanced search

#	Query	Results
1	Cochlear Implant*.mp. [mp=title, book title, abstract, original title, name of substance word, subject heading word, floating sub-heading word, keyword heading word, organism supplementary concept word, protocol supplementary concept word, rare disease supplementary concept word, unique identifier, synonyms, population supplementary concept word, anatomy supplementary concept word]	19,510
2	Cochlear Implantation/ not animal*.mp. [mp=title, book title, abstract, original title, name of substance word, subject heading word, floating sub-heading word, keyword heading word, organism supplementary concept word, protocol supplementary concept word, rare disease supplementary concept word, unique identifier, synonyms, population supplementary concept word, anatomy supplementary concept word]	8,339
3	Electrodes, Implanted/ not animal*.mp. [mp=title, book title, abstract, original title, name of substance word, subject heading word, floating sub-heading word, keyword heading word, organism supplementary concept word, protocol supplementary concept word, rare disease supplementary concept word, unique identifier, synonyms, population supplementary concept word, anatomy supplementary concept word]	12,136
4	(electroacoustic stimulation or electric acoustic implant*).mp. [mp=title, book title, abstract, original title, name of substance word, subject heading word, floating sub-heading word, keyword heading word, organism supplementary concept word, protocol supplementary concept word, rare disease supplementary concept word, unique identifier, synonyms, population supplementary concept word, anatomy supplementary concept word]	58
5	exp Adult/	7,857,304
6	(adult* or elderly or older people or older person or geriatric*).mp. [mp=title, book title, abstract, original title, name of substance word, subject heading word, floating sub-heading word, keyword heading word, organism supplementary concept word, protocol supplementary concept word, rare disease supplementary concept word, unique identifier, synonyms, population supplementary concept word, anatomy supplementary concept word]	6,679,673
7	5 or 6	8,722,849
8	1 or 2 or 3 or 4	30,874
9	7 and 8	15,231
10	patient care management/ or "delivery of health care"/ or "delivery of health care, integrated"/ or health services accessibility/ or healthcare disparities/ or managed care programs/ or telemedicine/ or remote consultation/ or telerehabilitation/ or patient selection/ or "quality of health care"/ or guideline adherence/ or patient outcome assessment/ or patient reported outcome measures/ or process assessment, health care/ or program evaluation/ or quality assurance, health care/ or quality improvement/ or quality indicators, health care/ or health care costs/ or "health services needs and demand"/ or professional practice gaps/ or professional-patient relations/ or clinical competence/	776,633
11	((service adj3 delivery) or (service adj3 provision) or candidacy* or selection criteria or referral*).mp. [mp=title, book title, abstract, original title, name of substance word, subject heading word, floating sub-heading word, keyword heading word, organism supplementary concept word, protocol supplementary concept word, rare disease supplementary concept word, unique identifier, synonyms, population supplementary concept word, anatomy supplementary concept word]	247,649

12	(((postoperative* adj3 performance) or (surg* adj3 technique) or (hearing adj3 preservation) or soft surg* or (surg* adj3 complication) or (postoperative adj3 complication) or (postsurg* adj3 complication) or (cochlea* adj1 trauma)) not animal*).mp. [mp=title, book title, abstract, original title, name of substance word, subject heading word, floating sub-heading word, keyword heading word, organism supplementary concept word, protocol supplementary concept word, rare disease supplementary concept word, unique identifier, synonyms, population supplementary concept word, anatomy supplementary concept word]	81,132
13	(electrode adj1 arra*).mp. [mp=title, book title, abstract, original title, name of substance word, subject heading word, floating sub-heading word, keyword heading word, organism supplementary concept word, protocol supplementary concept word, rare disease supplementary concept word, unique identifier, synonyms, population supplementary concept word, anatomy supplementary concept word]	6,124
14	((postlingual* or post-lingual* or pre-impl* or pre impl* or post impl* or post-impl* or residual) adj3 hear*).mp. [mp=title, book title, abstract, original title, name of substance word, subject heading word, floating sub-heading word, keyword heading word, organism supplementary concept word, protocol supplementary concept word, rare disease supplementary concept word, unique identifier, synonyms, population supplementary concept word, anatomy supplementary concept word]	1,859
15	(speech process* or external process* or map* or (remote adj3 test*) or (remote adj3 fit*) or performance*).mp. [mp=title, book title, abstract, original title, name of substance word, subject heading word, floating sub-heading word, keyword heading word, organism supplementary concept word, protocol supplementary concept word, rare disease supplementary concept word, unique identifier, synonyms, population supplementary concept word, anatomy supplementary concept word]	2,073,679
16	(uptake or adoption* or utilisation* or access).mp. [mp=title, book title, abstract, original title, name of substance word, subject heading word, floating sub-heading word, keyword heading word, organism supplementary concept word, protocol supplementary concept word, rare disease supplementary concept word, unique identifier, synonyms, population supplementary concept word, anatomy supplementary concept word]	920,555
17	((handelling adj 1 skill*) or (device manag* adj3 skill*)).mp. [mp=title, book title, abstract, original title, name of substance word, subject heading word, floating sub-heading word, keyword heading word, organism supplementary concept word, protocol supplementary concept word, rare disease supplementary concept word, unique identifier, synonyms, population supplementary concept word, anatomy supplementary concept word]	4
18	((postoperative adj2 outcome*) or (post-operative adj2 outcome*) or (post sur* adj3 outcome*)).mp. [mp=title, book title, abstract, original title, name of substance word, subject heading word, floating sub-heading word, keyword heading word, organism supplementary concept word, protocol supplementary concept word, rare disease supplementary concept word, unique identifier, synonyms, population supplementary concept word, anatomy supplementary concept word]	30,454
19	10 or 11 or 12 or 13 or 14 or 15 or 16 or 17 or 18	3,851,127
20	9 and 19	5,978
21	limit 20 to (english language and yr="2000 - 2022")	4,959

Database: Ovid Embase January 2000 to June 2022
Advanced search

#	Query	Results
1	Cochlear Implant*.mp. [mp=title, abstract, heading word, drug trade name, original title, device manufacturer, drug manufacturer, device trade name, keyword heading word, floating subheading word, candidate term word]	21,481
2	Cochlear Implantation/ not animal*.mp. [mp=title, abstract, heading word, drug trade name, original title, device manufacturer, drug manufacturer, device trade name, keyword heading word, floating subheading word, candidate term word]	6,081
3	Electrodes, Implanted/ not animal*.mp. [mp=title, abstract, heading word, drug trade name, original title, device manufacturer, drug manufacturer, device trade name, keyword heading word, floating subheading word, candidate term word]	1,325
4	(electroacoustic stimulation or electric acoustic implant*).mp. [mp=title, abstract, heading word, drug trade name, original title, device manufacturer, drug manufacturer, device trade name, keyword heading word, floating subheading word, candidate term word]	83
5	exp Adult/	10,647,573
6	(adult* or elderly or older people or older person or geriatric*).mp. [mp=title, abstract, heading word, drug trade name, original title, device manufacturer, drug manufacturer, device trade name, keyword heading word, floating subheading word, candidate term word]	10,518,155
7	5 or 6	11,485,895
8	1 or 2 or 3 or 4	22,590
9	7 and 8	10,351
10	patient care management/ or "delivery of health care"/ or "delivery of health care, integrated"/ or health services accessibility/ or healthcare disparities/ or managed care programs/ or telemedicine/ or remote consultation/ or telerehabilitation/ or patient selection/ or "quality of health care"/ or guideline adherence/ or patient outcome assessment/ or patient reported outcome measures/ or process assessment, health care/ or program evaluation/ or quality assurance, health care/ or quality improvement/ or quality indicators, health care/ or health care costs/ or "health services needs and demand"/ or professional practice gaps/ or professional-patient relations/ or clinical competence/	2,079,735
11	((service adj3 delivery) or (service adj3 provision) or candidacy* or selection criteria or referral*).mp. [mp=title, abstract, heading word, drug trade name, original title, device manufacturer, drug manufacturer, device trade name, keyword heading word, floating subheading word, candidate term word]	388,570
12	(((postoperative* adj3 performance) or (surg* adj3 technique) or (hearing adj3 preservation) or soft surg* or (surg* adj3 complication) or (postoperative adj3 complication) or (postsurg* adj3 complication) or (cochlea* adj1 trauma)) not animal*).mp. [mp=title, abstract, heading word, drug trade name, original title, device manufacturer, drug manufacturer, device trade name, keyword heading word, floating subheading word, candidate term word]	755,062
13	(electrode adj1 arra*).mp. [mp=title, abstract, heading word, drug trade name, original title, device manufacturer, drug manufacturer, device trade name, keyword heading word, floating subheading word, candidate term word]	8,112
14	((postlingual* or post-lingual* or pre-impl* or pre impl* or post impl* or post-impl* or residual) adj3 hear*).mp. [mp=title, abstract, heading word, drug trade name, original title, device manufacturer, drug manufacturer, device trade name, keyword heading word, floating subheading word, candidate term word]	2,397
15	(speech process* or external process* or map* or (remote adj3 test*) or (remote adj3 fit*) or performance*).mp. [mp=title, abstract, heading word, drug trade name, original title, device manufacturer, drug manufacturer, device trade name, keyword heading word, floating subheading word, candidate term word]	2,810,823
16	(uptake or adoption* or utilisation* or access).mp. [mp=title, abstract, heading word, drug trade name, original title, device manufacturer, drug manufacturer, device trade name, keyword heading word, floating subheading word, candidate term word]	1,367,510

17	((handelling adj 1 skill*) or (device manag* adj3 skill*)).mp. [mp=title, abstract, heading word, drug trade name, original title, device manufacturer, drug manufacturer, device trade name, keyword heading word, floating subheading word, candidate term word]	4
18	((postoperative adj2 outcome*) or (post-operative adj2 outcome*) or (post sur* adj3 outcome*)).mp. [mp=title, abstract, heading word, drug trade name, original title, device manufacturer, drug manufacturer, device trade name, keyword heading word, floating subheading word, candidate term word]	49,062
19	10 or 11 or 12 or 13 or 14 or 15 or 16 or 17 or 18	6,707,020
20	9 and 19	5,984
21	limit 20 to (english language and yr="2000 - 2022")	5,195
