# Supplementary material for: Models of service delivery in adult cochlear implantation and evaluation of outcomes: A scoping review of delivery arrangements
Source: PLoS One. 2023 May 10;18(5):e0285443. doi: 10.1371/journal.pone.0285443 (PMC10171603; doi:10.1371/journal.pone.0285443)
Supplement: S3 File — (PDF) [file pone.0285443.s003.pdf]

Supplementary file 3

Delivery arrangement category			
**How and When care is delivered (n=150)**			
Sub-category	*Definition*	*Number of studies*	*Details*
**Queuing strategies**	A reduction or increase in time to access a healthcare intervention, for example managed waiting lists, managing ER wait time.	10	o Surgical duration for CIs and influencing factors (unilateral, bilateral, revision and re-implantation, obesity) (1-3)<br>o Same day cochlear implant consultation and implantation; patient satisfaction with the model (4, 5)<br>o Early activation of CIs (6, 7)<br>o Impact of surgical waiting time on psychosocial wellbeing of CI candidates (8)<br>o Impact of inter-implant intervals (9)<br>o Reducing time to access pre and post-op (10)
**Quality and safety systems**	Essential standards for quality of healthcare, and reduction of poor outcomes related to unsafe healthcare.	137	o Safety profile of CIs : post-op infection incidence, timing and management (11-28) ; vestibular and balance disturbance, tinnitus (29-58); device failure (59); tip fold-over (60); taste disturbance (61-64); electrode translocation (65);anaesthetics (66, 67); CI in specific cases (68-70); Safety profile of diathermy in CI recipients (71) and patients' view (72); electrode deactivation (73)<br>o MRI safety in cochlear implant recipients (74-78)<br>o Interventions to quality control intra-cochlear electrode positioning peri and post-op (X-Ray, rotational tomography, co-registered cone beam CT scan and MRI, cone bean CT scan, flat panel CT scan, ECochG and CT scan, fluoroscopy) (79-92)<br>o Interventions to reduce post cochlear implant morbidities (antibiotics, skin flap and magnet displacement management, minimal hair shave, facial nerve palsy, pain management and opioids) (93-104)<br>o Adherence to pre-op immunisation guidelines (105-108)<br>o Interventions to improve surgical outcomes (application of steroids, under water surgical techniques, Co2 laser assisted surgery) (109-116)<br>o Diagnostic utility of pre-op imaging in surgical management decision-making (117, 118)<br>o Surgical approach to improve safety and efficiency (119)<br>o Interventions to evaluate and manage vestibular damage peri and post cochlear implantation (utility of vHIT, vestibular rehabilitation, application of VEMP) (120-122)<br>o Revision CI surgery and re-implantation to manage complications (123-133)<br>o Interventions to manage non-auditory stimulation (134-137)<br>o Interventions to manage hard-failure (138-142)<br>o Interventions to diagnose, reduce or improve electrode migration (143-147)
**Triage**	Management of patients attending a healthcare facility, or contacting a healthcare professional by phone, and receiving advice or being referral to an appropriate service.	3	o Improving patient flow (Same day triage system model) and patients' satisfaction with the model (148, 149)<br>o Delayed follow up due to COVID-19 (150)
**Where care is provided and changes to the healthcare environment (n=2)**			
Sub-category	*Definition*	*Number of studies*	*Details*
**Site of service delivery**	Changes in where care is provided, for example home vs. healthcare facility, inpatient vs.	2	o Programming of CI in decentralized private clinics (151)<br>o Utility of testing for candidacy in community clinics (152)

	outpatient, specialized vs. non-specialized facility, walk in clinics, medical day hospital, mobile units		

Who provides care and how the healthcare workforce is managed. (n= 11)

Sub-category	*Definition*	*Number of studies*	*Details*
**Role expansion of task shifting**	Expanding tasks undertaken by a cadre of health workers or shifting tasks from one cadre to another, to include tasks not previously part of their scope of practice.	1	○ Psychosocial counseling skills for audiologists (153)
**Self-management**	Shifting or promoting the responsibility for healthcare or disease management to the patient and/or their family.	7	○ Self- assessment and home-based evaluation of post-operative progress in CI recipients (154-157)<br>○ Self-programming of CI external processors (158, 159)<br>○ Self-help cognitive behavioral therapy program (160)
**Length of consultation**	Changes in the length of consultations.	1	○ Faster map generation in an appointment (161)
**Pre-licensure education**	Changes in pre-licensure education of health professionals.	2	○ Postgraduate specialisation fellowship for audiologists (162)<br>○ Intervention to improve counseling skills (Narrative competence) (163)

Coordination of care and management of care processes (n= 68)

Sub-category	*Definition*	*Number of studies (n)*	*Details*
**Care pathway**	Aim to link evidence to practice for specific health conditions and local arrangements for delivering care.	16	○ Remote follow up pathway for cochlear implant recipients (164, 165)<br>○ Clinical care pathway for patients with SSD (166)<br>○ Anesthetics care pathway for cochlear implantation; local vs general (Safety, cost, effectiveness, patient satisfaction) (167-171)<br>○ Evidence-based cochlear implant selection criteria (172-178)<br>○ Comprehensive self- administered CI selection test (179)
**Comprehensive geriatric assessment**	A multidimensional interdisciplinary diagnostic process focused on determining a frail older person's medical, psychological and functional capability to ensure that problems are identified, quantified and managed appropriately.	1	○ Improving assessment of elderly in otolaryngology clinics, physical performance battery (180)
**Disease management**	Programs designed to manage or prevent a chronic condition using a systematic approach to care and potentially employing multiple ways of influencing patients, providers or the process of care.	11	○ Hearing management in patients with head trauma (181-183)<br>○ Management of patients with NF2 with CI: decision making tool for CI vs ABI (184); CI without tumour removal (185, 186); comparison of CI outcome with and without tumour removal (187); Comparison of CI outcome in irradiated and non-irradiated ears (188-190); CI in unilateral vestibular Schwannoma (191)
**Packages of care**	Introduction, modification, or	32	○ Alternative test materials for testing patients with CI: AB words test as a candidacy test (192); non-linguistic tests for candidacy

	removal of packages of services designed to be implemented together for a particular diagnosis/disease, e.g. tuberculosis management guidelines, newborn care protocols.		(193-195); non-linguistic tests to follow up progress (196); using TEN test for CI eligibility (197)<br>o Use of objective measures to assist CI fitting: use of aided CAEP in SSD CI users (198); image-guided maps in CI users (IGCIP) (199, 200), image-based electrode deactivation reprogramming technique (IBEDRT) (201)<br>o Use of auditory and communication training packages to improve outcome post-CI: use of intensive psychophysical auditory training (202); auditory verbal skill training (AVST) (203, 204); combination of speech and sign therapy (Sim-Com) for improving communication in noise (205); communication strategy therapy in older adults (206); Digit in noise training (207);modulated telephone signal for telephone rehabilitation therapy (208); structured group-based therapy communication program (209); Phoneme training in older adults (210, 211)<br>o Music therapy program: Individual, face-to face (212); computer-based verbal and visual therapy (213, 214); web-based instrument recognition therapy (215); gamified AVT to improve speech in noise (216)<br>o Use of objective measures to improve decision making in cochlear implantation: prognostic value of fNIRS for CI outcome (217), prognostic value of radiodensity in measurement of cochlear ossification and fibrosis (218); scoring system for CI candidacy in VS (219); use of imaging to measure skin flap (220)<br>o Robot-assisted electrode insertion (221)<br>o Utility of ultrasound in diagnosis of magnet dislocation (222)<br>o Vestibular rehabilitation to improve balance function (223)
Patient-initiated appointment system	Systems that enable patients to make urgent appointments when they feel they cannot manage their condition or where something has changed unexpectedly.	1	o Traditional vs. patient-led postoperative review appointments (224)
Procurement and distribution of supplies	Systems for procuring and distributing drugs or other supplies.	3	o Impact of financial incentives in cochlear implant access (225)<br>o Impact of Medicaid on cochlear implant access (USA) (226)<br>o Impact of surgical markup on access (227)
Referral system	Systems for managing referrals of patients between health care providers	2	o Cochlear implant referrals from hearing aid to cochlear implant clinics (228)<br>o Intervention to improve cochlear implant referrals from hearing aid audiologists (229)
Shared decision making	Sharing healthcare decision making responsibilities among different individuals, potentially including the patient.	1	o Agreement of cochlear implantation success between cochlear implant recipients and significant others (230)
Teams	Creating and delivering care through a multidisciplinary team of healthcare workers.		o Shared Medical Appointments (231)
**Information and communication technology (ICT) (n=49)**			
Sub-category	*Definition*	*Number of studies (n)*	*Details*
Health information system	Health record and health management systems to store and manage patient health information, for example electronic patient records, or systems for recalling patients for follow-up or	11	o Long term follow up of CIs through a national and international databases: Function, device use and complications (232); Adverse events (233); MRI complications (234); long-term f/up planning (235)<br>o Digitisation of the ENT health records for CI patients (236)<br>o Digital multi-faceted protocol to improve pneumococcal vaccination rate in hospitals (237)<br>o Use of a national CI registry and single-center databases to determine CI candidacy (238-242)

	prevention e.g., immunization.		
**The use of information and communication technology**	Technology based methods to transfer healthcare information and support the delivery of care.	14	o Use of VR in training (ENT registrars trained for CI surgery) (243, 244)<br>o A tablet-based tool to assist surgeons in electrode insertion (245)<br>o Digital awareness campaign for CI in older adults (246)<br>o Modelling data and data mining: screening tool to identify CI candidates (247, 248); screening tool to identify second side CI candidates (249)<br>o Image guided mapping at a distant site (250)<br>o Machine learning and automated changes in maps: Using FOX2 software (251); FOX software (252, 253); Machine learning and postoperative outcome prediction (254)<br>o Multimedia digital support tool to educate potential CI candidates (255)<br>o Web-based information for consumers about CI (256)
**Smart home technologies**	Electronic assistive technologies.	20	o Web-base at home auditory training packages: phonemes and words (257); music training (The Hear Tunes software) (258)<br>o Wireless home technologies: phone clip to improve understanding on phones by CI users (259), bimodal users (260); CROS MIC to improve speech in noise understanding and localisation in unilateral CI users (261-267), in bilateral users (268, 269); remote MIC to enhance speech understanding in noise in bimodal users (270); use of Roger FM system in SSD CI users (271, 272); to improve speech understanding on TV (273)<br>o Smart phone application for tinnitus relief in CI users (274, 275), to assess CI progress (276)
**Telemedicine**	Exchange of healthcare information from one site to another via electronic communication.	4	o Remote programming of CIs external processor (277-279)<br>o Telemedicine for postoperative care (280)

Studies / interventions with specific goals (n= 77)

Sub-category	*Definition*	*Number of studies (n)*	*Details*
**Current practices in hearing healthcare to manage potential and existing CI recipients (Candidacy and referral, fitting, surgical choice).**		19	o Current practices and attitude in CI programming in audiology clinics: bimodal fitting (281-283); mapping of the external processor (284)<br>o Current practices and knowledge and attitude of CI audiological and surgical candidacy assessment: in ENT surgeons (285-288); Non-ENT surgeons (289);ENT surgeons providing CI services to humanitarian programs (290); audiologists (291, 292); second side CI candidacy (293, 294); international differences in candidacy and recommendations (295)<br>o Current practices of primary care physicians in CI referrals (296)<br>o Knowledge and current practice of vocational Rehabilitation Counsellors about CI (297)<br>o Current service provision to older adult CI candidates and recipients (298, 299)
**Economic analysis of CI services: cost analysis of CIs, auditory training and pre-op imaging; cost utility analysis; cost effectiveness analysis of CIs, and impact of CI on the income and employment of recipients.**		20	o Cost- effectiveness of CI: unilateral CI in public setting (300-303); impact of age on cost effectiveness of CIs compared to hearing aids in high income countries (304)<br>o Cost analysis of CI: surgical and first year rehabilitation cost of CI in France (305); sequential vs. simultaneous CIs in USA (306); life time cost of unilateral CI in adults in Germany (307); pre-operative imaging cost in post-lingual adults (308); cost analysis of various modes of auditory training (309)<br>o Cost utility analysis of CIs: bilateral CIs (310, 311), long term costs of bilateral CIs in publicly funded setting (312), simultaneous bilateral from insurance perspective (313); unilateral CI (314); SSD (315)<br>o Personal economics and societal benefit of CI for recipients (316, 317); societal factors (318);Employment and employment

		retention in CI recipients (319)
**Population or individual-based epidemiological studies: prevalence of CI in adults, hearing and socioeconomics characteristics of CI candidates and recipients, rates of CI uptake, device use and utilisation of healthcare.**	33	o Prevalence of CI in adult: prevalence of CIs in postlingually deafened adults in Sweden (320); prevalence of CI in Europe (321, 322); prevalence of CI and EAS in Japan (323); prevalence of CI in elderly in public system in USA (324); Prevalence and characteristics of hearing management in the USA (325); prevalence of severe to profound HL and CI in Sweden (326); prevalence and marker growth in USA (327)<br>o Hearing and socioeconomic profile of CI adult candidates and recipients: hearing profile in the USA (328, 329); hearing profile and service trends in Canada (330); socioeconomics and equality profile comparison between urban and rural areas in the USA (331); disparity in utilisation in USA (332); SSD (333)<br>o Device use in CI candidates and recipients: rate of hearing aid use in the non-implanted ear and influencing factors (334, 335); rate of hearing aid use in CI candidates and correlation with the uptake of CI (336); rate and cause of elective CI non-use amongst CI recipients (337, 338); CI use and satisfaction (339, 340)<br>o Rate of CI uptake: rate and correlation with demographic and socioeconomic factors (341, 342); rate of uptake and patients' perspective for non-adoption (343-347), audiometric configuration and uptake (348); racial disparity (349); profile and catchment (350)<br>o Rates of healthcare utilisation and subsequent management in elderly post-CI: short term post-CI compared to younger adults (351); long term audiological service utilisation and management (352)
**Patients' awareness and attitude about hearing and tinnitus management.**	4	o Awareness of and attitude towards HL management in older adults (353); Attitude and acceptance of invasive treatments for tinnitus amongst patients (354); knowledge and attitude about MRI (355); public awareness and attitude about CI(356)
QoL of caregivers	1	o QoL of caregivers to CI recipients (357)

References

1. Bennett ML, Sweeney AD, Haynes DS, Dietrich MS, Fang N, Labadie RF. The Utility of a Predictive Model for Cochlear Implant Operating Time. Otology & neurotology : official publication of the American Otological Society, American Neurotology Society [and] European Academy of Otology and Neurotology. 2016;37(2):e104-e9.
2. Hatch JL, Boersma IM, Weir FW, Bauschard MJ, Holcomb MA, Lambert PR, et al. The influence of obesity on operating room time and perioperative complications in cochlear implantation. World Journal of Otorhinolaryngology - Head and Neck Surgery.3(4):231-4.
3. Semaan MT, Fredman ET, Shah JR, Fares SA, Murray GS, Megerian CA. Surgical duration of cochlear implantation in an academic university-based practice. American Journal of Otolaryngology - Head and Neck Medicine and Surgery. 2013;34(5):382-7.
4. Nassiri AM, Yawn RJ, Gifford RH, Holder JT, Stimson CJ, Eavey RD, et al. Same-Day Patient Consultation and Cochlear Implantation: Innovations in Patient-Centered Health Care Delivery. Otology & neurotology : official publication of the American Otological Society, American Neurotology Society [and] European Academy of Otology and Neurotology. 2019;41(2):e223-e6.
5. Briggs J, Fields S, Smith C. Offering a Quality Service: Adult Patient Experience of Assessment for Cochlear Implantation within the 18 Week Pathway. Cochlear Implants International. 2010;11(sup2):25-30.
6. Gunther S, Baumann U, Stover T. Early Fitting in Cochlear Implantation: Benefits and Limits. Otology & neurotology : official publication of the American Otological Society, American Neurotology Society [and] European Academy of Otology and Neurotology. 2018;39(4):e250-e6.
7. Bruschke S, Baumann U, Stover T. Long-Term Follow-Up of Early Cochlear Implant Device Activation. Audiology & neuro-otology. 2021;26(5):327-37.
8. Guitar K, Giles EP, Raymond B, Welch D. Health effects of cochlear implants. New Zealand Medical Journal. 2013;126(1375):9-26.
9. Koo TK, Bartels LJ, Allen KP, Danner CJ. The relationship of inter-implant time and hearing outcomes for bilateral cochlear implants. American Journal of Otolaryngology - Head and Neck Medicine and Surgery. 2020.
10. Sladen DP, Olund AP, Schmitt M, Benson B, DeJong MD, Dowling BP, et al. Application of Kaizen Principles to a Large Cochlear Implant Practice: A Continuous Quality Improvement Initiative at Mayo Clinic. Otology & Neurotology. 2019;40(6):e592-e9.

11. Lander DP, Durakovic N, Kallogjeri D, Jiramongkolchai P, Olsen MA, Piccirillo JF, et al. Incidence of Infectious Complications Following Cochlear Implantation in Children and Adults. JAMA: Journal of the American Medical Association. 2020;323(2):182-3.
12. Olsen LB, Larsen S, Wanscher JH, Faber CE, Jeppesen J. Postoperative infections following cochlear implant surgery. Acta Oto-Laryngologica (Supplement). 2018;138(10):956-60.
13. Trinidade A, Rowl, s G, Obholzer R, Lavy J, Trinidade A, et al. Late skin flap failure following cochlear implantation. Cochlear Implants International: An Interdisciplinary Journal. 2008;9(3):167-75.
14. Cunningham CD, 3rd, Slattery WH, 3rd, Luxford WM. Postoperative infection in cochlear implant patients. Otolaryngology - Head & Neck Surgery.131(1):109-14.
15. Vijendren A, Ajith A, Borsetto D, Tysome JR, Axon PR, Donnelly NP, et al. Cochlear Implant Infections and Outcomes: Experience From a Single Large Center. Otology & Neurotology. 2020;41(9):e1105-e10.
16. Weder S, Shaul C, Won A, O'Leary S, Briggs RJ, Wong A, et al. Management of Severe Cochlear Implant Infections-35 Years Clinical Experience. Otology & Neurotology. 2020;41(10):1341-9.
17. Binnetoglu A, Demir B, Batman C. Surgical complications of cochlear implantation: a 25-year retrospective analysis of cases in a tertiary academic center. European Archives of Oto Rhino Laryngology. 2020.
18. Chiesa Estomba CM, Rivera Schmitz T, Betances Reinoso FA, Dominguez Collado L, Estevez Garcia M, Lorenzo Lorenzo AI. Complications after cochlear implantation in adult patients. 10-Year retrospective analysis of a tertiary academic centre. Auris, Nasus, Larynx.44(1):40-5.
19. Green KMJ, Bhatt YM, Saeed SR, Ramsden RT. Complications following adult cochlear implantation: Experience in Manchester. Journal of Laryngology and Otology.118(6):417-20.
20. Petersen H, Walshe P, Glynn F, McMahon R, Fitzgerald C, Thapa J, et al. Occurrence of major complications after cochlear implant surgery in Ireland. Cochlear Implants International: An Interdisciplinary Journal. 2018;19(6):297-306.
21. Farinetti A, Ben Gharbia D, Mancini J, Roman S, Nicollas R, Triglia JM. Cochlear implant complications in 403 patients: Comparative study of adults and children and review of the literature. European Annals of Otorhinolaryngology, Head and Neck Diseases.131(3):177-82.
22. Chen DS, Clarrett DM, Li L, Bowditch SP, Niparko JK, Lin FR. Cochlear implantation in older adults: long-term analysis of complications and device survival in a consecutive series. Otology & neurotology : official publication of the American Otological Society, American Neurotology Society [and] European Academy of Otology and Neurotology. 2013;34(7):1272-7.
23. Stamatiou GA, Kyrodimos E, Sismanis A. Complications of cochlear implantation in adults. Annals of Otology, Rhinology and Laryngology.120(7):428-32.
24. Dutt SN, Ray J, Hadjihannas E, Cooper H, Donaldson I, Proops DW. Medical and surgical complications of the second 100 adult cochlear implant patients in Birmingham. Journal of Laryngology & Otology. 2005;119(10):759-64.
25. Hansen S, Anthonsen K, Stangerup S, Jensen JH, Thomsen J, Cayé-Thomasen P. Unexpected findings and surgical complications in 505 consecutive cochlear implantations: a proposal for reporting consensus. Acta Oto-Laryngologica. 2010;130(5):540-9.
26. Ikeya J, Kawano A, Nishiyama N, Kawaguchi S, Hagiwara A, Suzuki M. Long-term complications after cochlear implantation. Auris Nasus Larynx.40(6):525-9.
27. Gordon SA, Aylward A, Patel NS, Bowers C, Presson AP, Smith KR, et al. Does Frailty or Age Increase the Risk of Postoperative Complications Following Cochlear Implantation? OTO open. 2021;5(3):2473974X211044084.
28. Hammond-Kenny A, Borsetto D, Manjaly JG, Panova T, Vijendren A, Bance M, et al. Cochlear Implantation in Elderly Patients: Survival Duration, Hearing Outcomes, Complication Rates, and Cost Utility. Audiology & neuro-otology. 2022;27(2):156-65.
29. Coordes A, Basta D, Gotze R, Scholz S, Seidl RO, Ernst A, et al. Sound-induced vertigo after cochlear implantation. Otology & neurotology : official publication of the American Otological Society, American Neurotology Society [and] European Academy of Otology and Neurotology.33(3):335-42.
30. Enticott JC, Tari S, Koh SM, Dowell RC, O'Leary SJ. Cochlear implant and vestibular function. Otology and Neurotology.27(6):824-30.
31. Filipo R, Patrizi M, La Gamma R, D'Elia C, La Rosa G, Barbara M. Vestibular impairment and cochlear implantation. Acta Oto-Laryngologica. 2006;126(12):1266-74.
32. Fina M, Skinner M, Goebel JA, Piccirillo JF, Neely JG. Vestibular dysfunction after cochlear implantation. Otology and Neurotology.24(2):234-42.
33. le Nobel GJ, Euna H, Wu A, Cushing S, Lin VY. Vestibular function following unilateral cochlear implantation for profound sensorineural hearing loss. Journal of Otolaryngology -- Head & Neck Surgery. 2016;45:1-6.
34. Meli A, Aud BM, Aud ST, Aud RG, Cristofari E. Vestibular function after cochlear implant surgery. Cochlear Implants Int. 2016;17(3):151-7.
35. Krause E, Louza JP, Hempel JM, Wechtenbruch J, Rader T, Gurkov R. Effect of cochlear implantation on horizontal semicircular canal function. European Archives of Oto-Rhino-Laryngology.266(6):811-7.
36. Melvin TA, Della Santina CC, Carey JP, Migliaccio AA, Melvin T-AN, Della Santina CC, et al. The effects of cochlear implantation on vestibular function. Otology & Neurotology. 2009;30(1):87-94.
37. Murray D, Viani L, Garvan J, Murphy A, ra, Vance R, et al. Balance, gait and dizziness in adult cochlear implant users: A cross sectional study. Cochlear Implants International.21(1):46-52.

38. Tsukada K, Moteki H, Fukuoka H, Iwasaki S, Usami S-i. Effects of EAS cochlear implantation surgery on vestibular function. Acta Oto-Laryngologica. 2013;133(11):1128-32.
39. Veroul E, Sabban D, Blexmann L, Frachet B, Poncet-Wallet C, Mamelle E. Predictive factors of vertigo following cochlear implantation in adults. European Archives of Oto Rhino Laryngology. 2020;04:04.
40. Frodlund J, Harder H, Maki-Torkko E, Ledin T. Vestibular Function After Cochlear Implantation: A Comparison of Three Types of Electrodes. Otology & neurotology : official publication of the American Otological Society, American Neurotology Society [and] European Academy of Otology and Neurotology.37(10):1535-40.
41. Alzhrani F. Considerations to improve the quality of cochlear implant surgery using measurements on postoperatively measured changes in the vestibular system. Hearing, Balance and Communication. 2018;16(2):108-13.
42. Zawawi F, Alobaid F, Leroux T, Zeitouni AG. Patients reported outcome post-cochlear implantation: how severe is their dizziness? Journal of Otolaryngology: Head and Neck Surgery. 2014;43:49.
43. Louza J, Klappert CL, Ledderose G, Gurkov R, Krause E. Cochlear Implant Surgery and the Risk of Falls in an Adult Population. Otology & neurotology : official publication of the American Otological Society, American Neurotology Society [and] European Academy of Otology and Neurotology.39(2):e74-e9.
44. Stevens MN, Baudhuin JE, Hullar TE. Short-term risk of falling after cochlear implantation. Audiology & neuro-otology. 2014;19(6):370-7.
45. Wiszomirska I, Zdrodowska A, Tacikowska G, Sosna M, Kaczmarczyk K, Skarżyński H. Does cochlear implantation influence postural stability in patients with hearing loss? Gait & Posture. 2019;74:40-4.
46. Mikkelsen KS, Ovesen T, Swan CZ. Pre-and post-operative dizziness, tinnitus, and taste disturbances among cochlear implant recipients. Journal of Laryngology and Otology.131(4):309-15.
47. Stieger C, Siemens X, Honegger F, Roushan K, Bodmer D, Allum J. Balance Control during Stance and Gait after Cochlear Implant Surgery. Audiology & Neuro-Otology. 2018;23(3):165-72.
48. Limb CJ, Francis HF, Lustig LR, Niparko JK, Jammal H. Benign positional vertigo after cochlear implantation. Otolaryngol Head Neck Surg. 2005;132(5):741-5.
49. Viccaro M, Mancini P, La Gamma R, De Seta E, Covelli E, Filipo R. Positional vertigo and cochlear implantation. Otology and Neurotology.28(6):764-7.
50. Krause E, Louza JP, Wechtenbruch J, Hempel JM, Rader T, Gurkov R. Incidence and quality of vertigo symptoms after cochlear implantation. Journal of Laryngology & Otology.123(3):278-82.
51. Shute WG, McOwan B, O'Leary SJ, Szmulewicz D, O'Leary SJ. The Early Postoperative Effects of Cochlear Implantation on Horizontal Semicircular Canal Function. Otology & Neurotology. 2018;39(7):e524-e31.
52. Kubo T, Yamamoto KI, Iwaki T, Doi K, Tamura M. Different forms of dizziness occurring after cochlear implant. European Archives of Oto-Rhino-Laryngology. 2001;258(1):9-12.
53. Matin F, Krueger C, Avallone E, Rossberg W, Demyanchuk A, Guenther A, et al. Influence of the Electrode Array Design on Incidence of Vertigo Symptoms and Vestibular Function After Cochlear Implantation. Ear, nose, & throat journal. 2021:1455613211022075.
54. Nayak N, Kellermeyer B, Dornton L, Heyd C, Kim CS, Wazen JJ. Vestibular dysfunction in cochlear implant candidates: Prevalence and outcomes. American journal of otolaryngology. 2022;43(1):103171.
55. Weinmann C, Baumann U, Leinung M, Stover T, Helbig S. Vertigo Associated With Cochlear Implant Surgery: Correlation With Vertigo Diagnostic Result, Electrode Carrier, and Insertion Angle. Frontiers in neurology. 2021;12(101546899):663386.
56. West N, Tian L, Vang Petersen LK, Bille M, Klokker M, Caye-Thomasen P. Objective Vestibular Test Battery and Patient Reported Outcomes in Cochlear Implant Recipients. Otology & neurotology : official publication of the American Otological Society, American Neurotology Society [and] European Academy of Otology and Neurotology. 2021;42(4):e416-e24.
57. Grimm DR, Fakurnejad S, Alyono JC. Cochlear Implantation and Risk of Falls in Older Adults. Otolaryngology--head and neck surgery : official journal of American Academy of Otolaryngology-Head and Neck Surgery. 2022;167(3):531-6.
58. Rasmussen KMB, West N, Tian L, Caye-Thomasen P. Long-Term Vestibular Outcomes in Cochlear Implant Recipients. Frontiers in neurology. 2021;12(101546899):686681.
59. Venail F, Sicard M, Piron JP, Levi A, Artieres F, Uziel A, et al. Reliability and complications of 500 consecutive cochlear implantations. Archives of Otolaryngology -- Head & Neck Surgery.134(12):1276-81.
60. Zuniga MG, Rivas A, ro, Hedley-Williams A, Gifford RH, Dwyer R, et al. Tip Fold-over in Cochlear Implantation: Case Series. Otology & Neurotology. 2016;37(10):N.PAG-N.PAG.
61. Mueller CA, Khatib S, Temmel AFP, Baumgartner WD, Hummel T. Effects of cochlear implantation on gustatory function. Annals of Otology, Rhinology and Laryngology.116(7):498-501.
62. Walliczek-Dworschak U, Knauer CM, Murbe D, Mainka A, Hummel T. Analysis of taste function in patients before and after cochlear implant surgery. Rhinology.56(2):149-54.
63. Nordfalk KF, Rasmussen K, Hopp E, Bunne M, Silvola JT, Jablonski GE. Insertion Depth in Cochlear Implantation and Outcome in Residual Hearing and Vestibular Function. Ear and hearing.37(2):e129-e37.
64. Beutner D, Vent J, Seehawer J, Luers JC, Lang-Roth R, Wrobel C. Taste-strip gustometry in cochlear implanted patients. Laryngoscope investigative otolaryngology. 2021;6(3):496-502.

65. Liebscher T, Mewes A, Hoppe U, Hornung J, Brademann G, Hey M. Electrode Translocations in Perimodiolar Cochlear Implant Electrodes: Audiological and Electrophysiological Outcome. Zeitschrift fur Medizinische Physik. 2020;30:30.
66. Heilbronn C, Lin H, Bhattacharyya N. Adult ambulatory otologic surgery: Unplanned revisits and complications. Laryngoscope. 2019.
67. Coelho DH, Yeh J, Kim JT, Lalwani AK. Cochlear implantation is associated with minimal anesthetic risk in the elderly. Laryngoscope.119(2):355-8.
68. McKinnon BJ, Watts T. Subcutaneous emphysema and pneumolabyrinth plus pneumocephalus as complications of middle ear implant and cochlear implant surgery. ENT: Ear, Nose & Throat Journal. 2013;92(7):298-300.
69. Hunter JB, Carlson ML, Sweeney AD, Tombers NM, Wanna GB, Driscoll CLW, et al. Cochlear Implantation in the Setting of Perioperative Anticoagulation and Antiplatelet Therapy. Otolaryngology-Head & Neck Surgery. 2016;154(3):513-7.
70. Odabasi O, Mobley SR, Bolanos RA, Hodges A, Balkany T. Cochlear implantation in patients with compromised healing. Otolaryngology - Head and Neck Surgery. 2000;123(6):738-41.
71. Frampton SJ, Ismail-Koch H, Mitchell TE. How safe is diathermy in patients with cochlear implants? Ann R Coll Surg Engl. 2012;94(8):585-7.
72. Frampton SJ, Mitchell TE. Surgical safety issues relating to the use of diathermy in patients with cochlear implants: The patient's perspective. Cochlear Implants International: An Interdisciplinary Journal. 2014;15(1):48-52.
73. Wen C, Hwa TP, Kaufman AC, Brant JA, Eliades SJ, Bigelow DC, et al. Predictors of Postoperative Electrode Deactivation Among Adult Cochlear Implantees. Otology & neurotology : official publication of the American Otological Society, American Neurotology Society [and] European Academy of Otology and Neurotology. 2021;42(6):e675-e83.
74. Tam YC, Lee JWY, Gair J, Jackson C, Donnelly NP, Tysome JR, et al. Performing MRI Scans on Cochlear Implant and Auditory Brainstem Implant Recipients: Review of 14.5 Years Experience. Otology & neurotology : official publication of the American Otological Society, American Neurotology Society [and] European Academy of Otology and Neurotology.41(5):e556-e62.
75. Carlson ML, Neff BA, Link MJ, Lane JI, Watson RE, McGee KP, et al. Magnetic Resonance Imaging With Cochlear Implant Magnet in Place: Safety and Imaging Quality. Otology & neurotology : official publication of the American Otological Society, American Neurotology Society [and] European Academy of Otology and Neurotology.36(6):965-71.
76. Pross SE, Ward BK, Sharon JD, Weinreich HM, Nafi A, Francis HW, et al. A Prospective Study of Pain From Magnetic Resonance Imaging With Cochlear Implant Magnets In Situ. Otology & Neurotology. 2018;39(2):e80-e6.
77. Loth AG, Fischer K, Hey AK, Weis R, Leinung M, Burck I, et al. Magnetic Resonance Imaging in Patients With Hearing Implants - Follow-up on Prevalence and Complications. Otology & neurotology : official publication of the American Otological Society, American Neurotology Society [and] European Academy of Otology and Neurotology. 2021;42(9):1334-41.
78. Holtmann L, Hans S, Kaster F, Muller V, Lang S, Goricke S, et al. Magnet dislocation following magnetic resonance imaging in cochlear implant users: Diagnostic pathways and managment. Cochlear implants international. 2021;22(4):195-202.
79. Aschendorff A, Kubalek R, Turowski B, Zanella F, Hochmuth A, Schumacher M, et al. Quality control after cochlear implant surgery by means of rotational tomography. Otology and Neurotology.26(1):34-7.
80. Coombs A, Clamp PJ, Armstrong S, Robinson PJ, Hajioff D. The role of post-operative imaging in cochlear implant surgery: a review of 220 adult cases. Cochlear Implants International: An Interdisciplinary Journal. 2014;15(5):264-71.
81. Dragovic AS, Stringer AK, Campbell L, Shaul C, O'Leary SJ, Briggs RJ. Co-registration of cone beam CT and preoperative MRI for improved accuracy of electrode localization following cochlear implantation. Cochlear Implants International: An Interdisciplinary Journal. 2018;19(3):147-52.
82. Gleeson TG, Lacy PD, Bresnihan M, Gaffney R, Brennan P, Viani L. High resolution computed tomography and magnetic resonance imaging in the pre-operative assessment of cochlear implant patients. Journal of Laryngology and Otology.117(9):692-5.
83. Jia H, Torres R, Nguyen Y, De Seta D, Ferrary E, Wu H, et al. Intraoperative conebeam CT for assessment of intracochlear positioning of electrode arrays in adult recipients of cochlear implants. American Journal of Neuroradiology. 2018;39(4):768-74.
84. Arweiler-Harbeck D, Monninghoff C, Greve J, Hoffmann T, Goricke S, Arnolds J, et al. Imaging of Electrode Position after Cochlear Implantation with Flat Panel CT. Isrn Otolaryngology Print. 2012;2012:728205.
85. Jiam NT, Jiradejvong P, Pearl MS, Limb CJ. The Effect of Round Window vs Cochleostomy Surgical Approaches on Cochlear Implant Electrode Position: A Flat-Panel Computed Tomography Study. JAMA Otolaryngology-Head & Neck Surgery. 2016;142(9):873-80.
86. O'Connell BP, Holder JT, Dwyer RT, Gifford RH, Noble JH, Bennett ML, et al. Intra- and postoperative electrocochleography may be predictive of final electrode position and postoperative hearing preservation. Frontiers in Neuroscience. 2017;11.
87. Dalbert A, Huber A, Veraguth D, Roosli C, Pfiffner F. Assessment of Cochlear Trauma During Cochlear Implantation Using Electrocochleography and Cone Beam Computed Tomography. Otology & neurotology : official publication of the American Otological Society, American Neurotology Society [and] European Academy of Otology and Neurotology. 2016;37(5):446-53.

88. Perazzini C, Puechmaille M, Saroul N, Plainfosse O, Montrieul L, Becaud J, et al. Fluoroscopy guided electrode-array insertion for cochlear implantation with straight electrode-arrays: a valuable tool in most cases. European Archives of Oto Rhino Laryngology. 2020;25:25.
89. Keidar E, Singh J, Santiago-Rivera OJ, Wilkerson B, Babu S. Utility and value of pre-operative CT and MRI for cochlear implantation in the elderly. American journal of otolaryngology. 2021;42(3):102853.
90. Buechner A, Bardt M, Haumann S, Geissler G, Salcher R, Lenarz T. Clinical experiences with intraoperative electrocochleography in cochlear implant recipients and its potential to reduce insertion trauma and improve postoperative hearing preservation. PloS one. 2022;17(4):e0266077.
91. Lenarz T, Buechner A, Gantz B, Hansen M, Tejani VD, Labadie R, et al. Relationship Between Intraoperative Electrocochleography and Hearing Preservation. Otology & neurotology : official publication of the American Otological Society, American Neurotology Society [and] European Academy of Otology and Neurotology. 2022;43(1):e72-e8.
92. Klabbers TM, Huinck WJ, Mylanus EAM. Comparison Between Transimpedance Matrix (TIM) Measurement and X-ray Fluoroscopy for Intraoperative Electrode Array Tip Fold-Over Detection. Otology & neurotology : official publication of the American Otological Society, American Neurotology Society [and] European Academy of Otology and Neurotology. 2021;42(10):e1457-e63.
93. Gawecki W, Karlik M, Borucki L, Szyfter-Harris J, Wrobel M. Skin flap complications after cochlear implantations. European Archives of Oto-Rhino-Laryngology.273(12):4175-83.
94. Leonhard L, Roche J, Wiel, A, Pyle GM. The Temporoparietal Fascia Flap is an Effective Strategy for Cochlear Implant Wound Coverage. Annals of Otology, Rhinology and Laryngology.129(2):135-41.
95. Low WK, Rangabashyam M, Wang F. Management of major post-cochlear implant wound infections. European Archives of Oto-Rhino-Laryngology.271(9):2409-13.
96. Posner D, Scott A, Polite C, Lustig LR. External magnet displacement in cochlear implants: causes and management. Otology & neurotology : official publication of the American Otological Society, American Neurotology Society [and] European Academy of Otology and Neurotology.31(1):88-93.
97. Almosnino G, Zeitler DM, Schwartz SR. Postoperative Antibiotics Following Cochlear Implantation: Are They Necessary? Annals of Otology, Rhinology and Laryngology.127(4):266-9.
98. Alzhrani F, Lenarz T, Teschner M. Facial palsy following cochlear implantation. Eur Arch Otorhinolaryngol. 2016;273(12):4199-207.
99. Howard NS, Antonelli PJ. Complications of Cochlear Implant Placement with Minimal Hair Shave. American Journal of Otolaryngology - Head and Neck Medicine and Surgery.25(2):84-7.
100. Fayad JN, Wanna GB, Micheletto JN, Parisier SC. Facial nerve paralysis following cochlear implant surgery. Laryngoscope.113(8):1344-6.
101. Thom JJ, Carlson ML, Olson MD, Neff BA, Beatty CW, Facer GW, et al. The prevalence and clinical course of facial nerve paresis following cochlear implant surgery. Laryngoscope.123(4):1000-4.
102. Mahairas AD, Neff R, Craker N, McNulty BN, Shinn JB, Bush ML. Opioid Prescribing Patterns and Usage Following Cochlear Implantation. Otology & Neurotology. 2020;41(7):922-8.
103. Alnafjan F, Hasan Z, Sanli H, da Cruz MJ. Risk Factors for Facial Nerve and Other Nonauditory Side Effects Following Cochlear Implantation. Otology & neurotology : official publication of the American Otological Society, American Neurotology Society [and] European Academy of Otology and Neurotology. 2021;42(8):e1022-e9.
104. Dahm V, Lui JT, Liepins R, Chen JM, Le TN, Arnoldner C, et al. Is otologic surgery contributing to the opioid epidemic? Journal of otolaryngology - head & neck surgery = Le Journal d'oto-rhino-laryngologie et de chirurgie cervico-faciale. 2021;50(1):38.
105. Mitchell S, Nakhid-Schuster L, Neeff M. Adult cochlear implant recipients and meningitis in New Zealand: are patients receiving the recommended immunisations? New Zealand Medical Journal. 2020;133(1511):14-20.
106. Jeyakumar AS, Buchman CA, Jiang Y, McKinnon BJ. Cochlear implant provider awareness of vaccination guidelines. Otolaryngology - Head and Neck Surgery (United States).155:P105-P6.
107. Deb A, Mohanty S, Ou W, Rajagopalan S, Johnson KD. Pneumococcal vaccination coverage among adults aged 19 to 64 years with immuno-compromising conditions, cerebrospinal fluid (CSF) leaks, or cochlear implants in the US. Expert review of vaccines. 2021;20(3):331-45.
108. Loth A, Vazzana C, Leinung M, Guderian D, Issing C, Baumann U, et al. Quality control in cochlear implant therapy: clinical practice guidelines and registries in European countries. European archives of oto-rhino-laryngology : official journal of the European Federation of Oto-Rhino-Laryngological Societies (EUFOS) : affiliated with the German Society for Oto-Rhino-Laryngology - Head and Neck Surgery. 2022;279(10):4779-86.
109. Cho HS, Lee KY, Choi H, Jang JH, Lee SH. Dexamethasone Is One of the Factors Minimizing the Inner Ear Damage from Electrode Insertion in Cochlear Implantation. Audiology and neurotology. 2016;21(3):178-86.
110. Kuthubutheen J, Joglekar S, Smith L, Friesen L, Smilsky K, Millman T, et al. The Role of Preoperative Steroids for Hearing Preservation Cochlear Implantation: results of a Randomized Controlled Trial. Audiology and neurotology. 2018;22(4):292-302.
111. Enticott JC, Eastwood HT, Briggs RJ, Dowell RC, O'Leary SJ. Methylprednisolone applied directly to the round window reduces dizziness after cochlear implantation: a randomized clinical trial. Audiology & Neuro-Otology. 2011;16(5):289-303.

112. Rajan GP, Kuthubutheen J, Hedne N, Krishnaswamy J. The role of preoperative, intratympanic glucocorticoids for hearing preservation in cochlear implantation: A prospective clinical study. Laryngoscope.122(1):190-5.
113. Anagiotos A, Beutner D, Gostian A-O, Schwarz D, Luers J-C, Hüttenbrink K-B. Insertion of Cochlear Implant Electrode Array Using the Underwater Technique for Preserving Residual Hearing. Otology & Neurotology. 2016;37(4):339-44.
114. Stevens SM, Redmann A, Whitaker K, Ruotanen A, Houston L, Hammer T, et al. Preliminary Outcomes Report for CO2 Laser Assisted Electric-Acoustic Cochlear Implantation. Otology & neurotology : official publication of the American Otological Society, American Neurotology Society [and] European Academy of Otology and Neurotology. 2018;39(5):582-90.
115. Skarzynska MB, Kolodziejak A, Gos E, Skarzynski PH, Lorens A, Walkowiak A. The Clinical Effect of Steroid Therapy on Preserving Residual Hearing after Cochlear Implantation with the Advanced Bionics HiRes Ultra 3D Cochlear Implant System. Life (Basel, Switzerland). 2022;12(4).
116. Weder S, Bester C, Collins A, Shaul C, Briggs RJ, O'Leary S. Real Time Monitoring During Cochlear Implantation: Increasing the Accuracy of Predicting Residual Hearing Outcomes. Otology & neurotology : official publication of the American Otological Society, American Neurotology Society [and] European Academy of Otology and Neurotology. 2021;42(8):e1030-e6.
117. Schwartz SR, Chen BS. The role of preoperative imaging for cochlear implantation in postlingually deafened adults. Otology & neurotology : official publication of the American Otological Society, American Neurotology Society [and] European Academy of Otology and Neurotology. 2014;35(9):1536-40.
118. Tamplen M, Schwalje A, Lustig L, Alemi AS, Miller ME. Utility of preoperative computed tomography and magnetic resonance imaging in adult and pediatric cochlear implant candidates. Laryngoscope. 2016;126(6):1440-5.
119. Freni F, Gazia F, Slavutsky V, Scherdel EP, Nicenboim L, Posada R, et al. Cochlear Implant Surgery: Endomeatal Approach versus Posterior Tympanotomy. International Journal of Environmental Research & Public Health [Electronic Resource]. 2020;17(12):12.
120. Bittar RSM, Sato E, Ribeiro DJS, Oiticica J, Grasel SS, Mezzalira R, et al. Video head impulse test relevance in the early postoperative period after cochlear implantation. Acta Oto-Laryngologica. 2019;139(1):6-10.
121. Ernst A, Todt I, Seidl RO, Eisenschenk A, Blodow A, Basta D. The application of vestibular-evoked myogenic potentials in otoneurosurgery. Otolaryngology - Head and Neck Surgery.135(2):286-90.
122. Steenerson RL, Cronin GW, Gary LB. Vertigo after cochlear implantation. Otology and Neurotology. 2001;22(6):842-3.
123. Cote M, Ferron P, Bergeron F, Bussieres R. Cochlear reimplantation: Causes of failure, outcomes, and audiologic performance. Laryngoscope.117(7):1225-35.
124. Ray J, Proops D, Donaldson I, Fielden C, Cooper H. Explantation and reimplantation of cochlear implants. Cochlear Implants International. 2004;5(4):160-7.
125. Stevens SM, Dougherty H, Wenstrup L, Hammer T, Cole T, Redmann A, et al. Is Hard Failure Still a Common Indication for Revision Surgery in Adult Cochlear Implant Recipients? Otology & neurotology : official publication of the American Otological Society, American Neurotology Society [and] European Academy of Otology and Neurotology.40(3):321-7.
126. Sunde J, Webb JB, Moore PC, Gluth MB, Dornhoffer JL. Cochlear implant failure, revision, and reimplantation. Otology & neurotology : official publication of the American Otological Society, American Neurotology Society [and] European Academy of Otology and Neurotology.34(9):1670-4.
127. Brown KD, Connell SS, Balkany TJ, Eshraghi AE, Telischi FF, Angeli SA. Incidence and indications for revision cochlear implant surgery in adults and children. Laryngoscope. 2009;119(1):152-7.
128. Kimura KS, O'Connell BP, Nassiri AM, Dedmon MM, Haynes DS, Bennett ML. Outcomes of Revision Cochlear Implantation. Otology & Neurotology.41(6):e705-e11.
129. Buchman CA, Higgins CA, Cullen R, Pillsbury HC. Revision cochlear implant surgery in adult patients with suspected device malfunction. Otology and Neurotology.25(4):504-10.
130. Rivas A, Marlowe AL, Chinnici JE, Niparko JK, Francis HW. Revision cochlear implantation surgery in adults: Indications and results. Otology and Neurotology.29(5):639-48.
131. Hassepass F, Stabenau V, Maier W, Arndt S, Laszig R, Beck R, et al. Revision surgery due to magnet dislocation in cochlear implant patients: an emerging complication. Otology & neurotology : official publication of the American Otological Society, American Neurotology Society [and] European Academy of Otology and Neurotology.35(1):29-34.
132. Chen J, Chen B, Shi Y, Li Y. A retrospective review of cochlear implant revision surgery: a 24-year experience in China. European archives of oto-rhino-laryngology : official journal of the European Federation of Oto-Rhino-Laryngological Societies (EUFOS) : affiliated with the German Society for Oto-Rhino-Laryngology - Head and Neck Surgery. 2022;279(3):1211-20.
133. Sagiv D, Yaar-Soffer Y, Yakir Z, Henkin Y, Shapira Y. Rates, Indications, and Speech Perception Outcomes of Revision Cochlear Implantations. Journal of clinical medicine. 2021;10(15).
134. Broomfield S, Mawman D, Woolford TJ, O'Driscoll M, Luff D, Ramsden RT. Non-auditory stimulation in adult cochlear implant users. Cochlear Implants International.1(1):55-66.
135. Pires JS, Melo AS, Caiado R, Martins JH, Elói Moura J, Silva LF. Facial nerve stimulation after cochlear implantation: Our experience in 448 adult patients. Cochlear Implants International: An Interdisciplinary Journal. 2018;19(4):193-7.

136. Kaufman AC, Naples JG, Bigelow DC, Eliades SJ, Brant JA, Kaufman HS, et al. Lateral Wall Electrodes Increase the Rate of Postactivation Nonauditory Percepts. Otology & Neurotology. 2020;41(5):e575-e9.
137. Hatch JL, Rizk HG, Moore MW, Camposeo EE, Nguyen SA, Lambert PR, et al. Can Preoperative CT Scans Be Used to Predict Facial Nerve Stimulation Following CI? Otology & neurotology : official publication of the American Otological Society, American Neurotology Society [and] European Academy of Otology and Neurotology.38(8):1112-7.
138. Carlson ML, Archibald DJ, Dabade TS, Gifford RH, Neff BA, Beatty CW, et al. Prevalence and timing of individual cochlear implant electrode failures. Otology & neurotology : official publication of the American Otological Society, American Neurotology Society [and] European Academy of Otology and Neurotology.31(6):893-8.
139. Newbold C, Risi F, Hollow R, Yusof Y, Dowell R. Long-term electrode impedance changes and failure prevalence in cochlear implants. International Journal of Audiology. 2015;54(7):453-60.
140. Schow B, Friedl, DR, Jensen J, Burg L, Runge CL. Electrode failure and device failure in adult cochlear implantation. Cochlear Implants International: An Interdisciplinary Journal. 2012;13(1):35-40.
141. Maurer J, Marangos N, Ziegler E. Reliability of cochlear implants. Otolaryngology - Head and Neck Surgery.132(5):746-50.
142. Trotter MI, Backhouse S, Wagstaff S, Hollow R, Briggs RJ. Classification of cochlear implant failures and explantation: the Melbourne experience, 1982-2006. Cochlear implants international. 2009;10:105-10.
143. Dietz A, Wennstrom M, Lehtimaki A, Lopponen H, Valtonen H. Electrode migration after cochlear implant surgery: more common than expected? European Archives of Oto-Rhino-Laryngology.273(6):1411-8.
144. van der Marel KS, Verbist BM, Briaire JJ, Joemai RM, Frijns JH. Electrode migration in cochlear implant patients: not an exception. Audiology & neuro-otology. 2012;17(5):275-81.
145. Tange RA, Grolman W, Carelsen B. Migration of the ball electrode after cochlear implantation. Otology and Neurotology.28(2):195-8.
146. Rader T, Baumann U, Stover T, Weissgerber T, Adel Y, Leinung M, et al. Management of Cochlear Implant Electrode Migration. Otology & neurotology : official publication of the American Otological Society, American Neurotology Society [and] European Academy of Otology and Neurotology.37(9):e341-e8.
147. Leinung M, Helbig S, Adel Y, Stover T, Loth AG. The Effect of a Bone Groove Against Cochlear Implant Electrode Migration. Otology & neurotology : official publication of the American Otological Society, American Neurotology Society [and] European Academy of Otology and Neurotology. 2019;19.
148. Muff J. Patient satisfaction survey of the same day assessment for adult cochlear implant candidates. Cochlear Implants International. 2015;16:S36-9.
149. Harris F. Same day assessment for adult cochlear implant candidates. Cochlear Implants International.14:S52-S5.
150. Vos TG, Brown KD, Buss E, Bucker AL, Dedmon MM, O'Connell BP, et al. Influence of Postponed Follow-Up after Cochlear Implant Activation during the COVID-19 Pandemic on Aided Sound Field Detection and Speech Recognition. Audiology & neuro-otology. 2022;27(3):227-34.
151. Bourn S, Goldstein MR, Knickerbocker A, Jacob A. Decentralized Cochlear Implant Programming Network Improves Access, Maintains Quality, and Engenders High Patient Satisfaction. Otology & neurotology : official publication of the American Otological Society, American Neurotology Society [and] European Academy of Otology and Neurotology. 2021;42(8):1142-8.
152. Shah RR, Jeon JW, Naples JG, Hwa TP, Davis S, Eliades SJ, et al. Streamlining the Cochlear Implant Evaluation: Utility of Community Audiometry in Cochlear Implant Candidacy Assessment. Otology & neurotology : official publication of the American Otological Society, American Neurotology Society [and] European Academy of Otology and Neurotology. 2021;42(3):402-7.
153. Muñoz K, Ong CW, Borrie SA, Nelson LH, Twohig MP. Audiologists' communication behaviour during hearing device management appointments. International Journal of Audiology. 2017;56(5):328-36.
154. Cullington HE, Agyemang-Prempeh A. Person-centred cochlear implant care: Assessing the need for clinic intervention in adults with cochlear implants using a dual approach of an online speech recognition test and a questionnaire. Cochlear Implants International: An Interdisciplinary Journal. 2017;18(2):76-88.
155. de Graaff F, Huysmans E, Merkus P, Theo Goverts S, Smits C. Assessment of speech recognition abilities in quiet and in noise: a comparison between self-administered home testing and testing in the clinic for adult cochlear implant users. International Journal of Audiology. 2018;57(11):872-80.
156. de Graaff F, Huysmans E, Philips B, Merkus P, Goverts ST, Kramer SE, et al. Our experience with home self-assessment of speech recognition in the care pathway of 10 newly implanted adult cochlear implant users. Clinical Otolaryngology. 2019;44(3):446-51.
157. van Wieringen A, Magits S, Francart T, Wouters J. Home-Based Speech Perception Monitoring for Clinical Use With Cochlear Implant Users. Frontiers in neuroscience. 2021;15(101478481):773427.
158. Dwyer RT, Spahr T, Agrawal S, Hetlinger C, Holder JT, Gifford RH. Participant-generated Cochlear Implant Programs: Speech Recognition, Sound Quality, and Satisfaction. Otology & neurotology : official publication of the American Otological Society, American Neurotology Society [and] European Academy of Otology and Neurotology.37(7):e209-e16.
159. Vroegop JL, Dingemanse JG, van der Schroeff MP, Metselaar RM, Goedegebure A. Self-Adjustment of Upper Electrical Stimulation Levels in CI Programming and the Effect on Auditory Functioning. Ear and hearing.38(4):e232-e40.

160. Garnefski N, Kraaij V. Effects of a Cognitive Behavioral Self-help Program on Emotional Problems for People With Acquired Hearing Loss: A Randomized Controlled Trial. Journal of Deaf Studies & Deaf Education. 2012;17(1):75-84.
161. Kurz A, Hagen R, van de Heyning P, Mertens G. Flat-based fitting: the evaluation and usefulness of a new strategy-based fitting approach for cochlear implants. Eur Arch Otorhinolaryngol. 2019;276(11):3239-45.
162. Arenberg JG, Hull RH, Hunter L. Postgraduate Specialization Fellowship Training for Audiologists: Survey Results From Educators, Supervisors, and Students. American journal of audiology.1-10.
163. Berg AL, Canellas M, Salbod S, Velayo R. Exposure to disability and hearing loss narratives in undergraduate audiology curriculum. American journal of audiology. 2008;17(2):123-8.
164. Cullington H, Kitterick P, Weal M, Margol-Gromada M. Feasibility of personalised remote long-term follow-up of people with cochlear implants: a randomised controlled trial. BMJ open. 2018;8(4):e019640.
165. Holtmann LC, Deus E, Meyer M, Kaster F, Bastian T, Schleupner MC, et al. Detection accuracy of soft tissue complications during remote cochlear implant follow-up. Cochlear implants international. 2022;23(5):249-56.
166. Kurz A, Rak K, Hagen R, Ehrmann-Muller D. Evaluating the Decision for Cochlear Implantation in Individuals With Single-Sided Deafness (SSD); Implementing the SSD Consensus Protocol Into Clinical Routine. Otology & neurotology : official publication of the American Otological Society, American Neurotology Society [and] European Academy of Otology and Neurotology. 2020;14.
167. Pateron B, Bakhos D, Lelouarn A, Bordure P, Bozorg Grayeli A, Godey B, et al. Local anaesthesia and conscious sedation for cochlear implantation: Experience with 20 patients. Journal of Laryngology and Otology.130(2):151-6.
168. Toner F, Jackson CP, Toner JG. How we do it: Local anaesthetic cochlear implantation. Cochlear Implants International: An Interdisciplinary Journal. 2013;14(4):232-5.
169. Shabashev S, Fouad Y, Huncke TK, Rol, JT. Cochlear implantation under conscious sedation with local anesthesia; Safety, Efficacy, Costs, and Satisfaction. Cochlear Implants International: An Interdisciplinary Journal. 2017;18(6):297-303.
170. Hamerschmidt R, Moreira AT, Wiemes GR, Tenório SB, Tâmbara EM. Cochlear implant surgery with local anesthesia and sedation: comparison with general anesthesia. Otology & Neurotology. 2013;34(1):75-8.
171. Dietz A, Lenarz T. Cochlear implantation under local anesthesia in 117 cases: patients' subjective experience and outcomes. European archives of oto-rhino-laryngology : official journal of the European Federation of Oto-Rhino-Laryngological Societies (EUFOS) : affiliated with the German Society for Oto-Rhino-Laryngology - Head and Neck Surgery. 2022;279(7):3379-85.
172. Leigh JR, Moran M, Hollow R, Dowell RC. Evidence-based guidelines for recommending cochlear implantation for postlingually deafened adults. International Journal of Audiology. 2016;55:S3-S8.
173. Group UCIS. Criteria of Candidacy for Unilateral Cochlear Implantation in Postlingually Deafened Adults I: Theory and Measures of Effectiveness. Ear and Hearing. 2004;25(4):310-35.
174. Dowell RC, Hollow R, Winton E. Outcomes for cochlear implant users with significant residual hearing: implications for selection criteria in children. Archives of Otolaryngology -- Head & Neck Surgery.130(5):575-81.
175. Zwolan TA, Schvartz-Leyzac KC, Pleasant T. Development of a 60/60 Guideline for Referring Adults for a Traditional Cochlear Implant Candidacy Evaluation. Otology & Neurotology.41(7):895-900.
176. Hunter JB, Tolisano AM. When to Refer a Hearing-impaired Patient for a Cochlear Implant Evaluation. Otology & Neurotology Publish Ahead of Print. 2020;30:30.
177. Dornhoffer J, Liu YF, Zhao EE, Camposeo EL, Meyer TA, McRackan TR. Factors That Influence Second-side Cochlear Implant Speech Recognition Outcomes. Otology & neurotology : official publication of the American Otological Society, American Neurotology Society [and] European Academy of Otology and Neurotology. 2021;42(3):e279-e85.
178. Hunter JB, Tolisano AM. When to Refer a Hearing-impaired Patient for a Cochlear Implant Evaluation. Otology & neurotology : official publication of the American Otological Society, American Neurotology Society [and] European Academy of Otology and Neurotology. 2021;42(5):e530-e5.
179. Shafiro V, Hebb M, Walker C, Oh J, Hsiao Y, Brown K, et al. Development of the Basic Auditory Skills Evaluation Battery for Online Testing of Cochlear Implant Listeners. American Journal of Audiology.29(3S):577-90.
180. Chen DS, Blake CR, Genther DJ, Li L, Lin FR. Assessing physical functioning in otolaryngology: feasibility of the Short Physical Performance Battery. American Journal of Otolaryngology. 2014;35(6):708-12.
181. Alves M, Martins JH, Moura JE, Ramos D, Alves H, Oliveira G, et al. Auditory rehabilitation after cochlear implantation in adults with hearing impairment after head trauma. Cochlear Implants International: An Interdisciplinary Journal. 2014;15(6):312-7.
182. Greenberg SL, Shipp D, Lin VY, Chen JM, Nedzelski JM. Cochlear implantation in patients with bilateral severe sensorineural hearing loss after major blunt head trauma. Otology & neurotology : official publication of the American Otological Society, American Neurotology Society [and] European Academy of Otology and Neurotology.32(1):48-54.
183. Khwaja S, Mawman D, Nichani J, Bruce I, Green K, Lloyd S. Cochlear implantation in patients profoundly deafened after head injury. Otology & Neurotology. 2012;33(8):1328-32.
184. Harris F, Tysome JR, Donnelly N, Durie-Gair J, Crundwell G, Tam YC, et al. Cochlear implants in the management of hearing loss in Neurofibromatosis Type 2. Cochlear Implants International: An Interdisciplinary Journal. 2017;18(3):171-9.
185. Mukherjee P, Ramsden JD, Donnelly N, Axon P, Saeed S, Fagan P, et al. Cochlear implants to treat deafness caused by vestibular schwannomas. Otology & neurotology : official publication of the American Otological Society, American Neurotology Society [and] European Academy of Otology and Neurotology.34(7):1291-8.

186. Tan H, Jia H, Li Y, Zhang Z, Zhu W, Cai Y, et al. Impact of cochlear implantation on the management strategy of patients with neurofibromatosis type 2. European Archives of Oto-Rhino-Laryngology.275(11):2667-74.
187. North HJD, Mawman D, O'Driscoll M, Freeman SR, Rutherford SA, King AT, et al. Outcomes of cochlear implantation in patients with neurofibromatosis type 2. Cochlear Implants International: An Interdisciplinary Journal. 2016;17(4):172-7.
188. Patel NS, Link MJ, Neff BA, Carlson ML, Driscoll CL. Cochlear implantation after radiosurgery for vestibular schwannoma. Journal of Neurological Surgery, Part B Skull Base Conference: 30th Annual Meeting North American Skull Base Society United States. 2020;81.
189. Soh JM, D'Souza VD, Sarepaka GK, Ng WN, Ong CS, Low WK. Cochlear Implant Outcomes: A Comparison between Irradiated and Non-irradiated Ears. Clin Exp Otorhinolaryngol.5:S93-S8.
190. Chang WW, Yeung KN, Luk BP, Leung KK, Sung JK, Tong MC. Cochlear implantation in postirradiated ears: A case-control comparative study. Laryngoscope Investigative Otolaryngology.5(6):1163-7.
191. Sanna M, Medina MdM, Macak a, Rossi G, Sozzi V, Prasad SC, et al. Vestibular Schwannoma Resection with Ipsilateral Simultaneous Cochlear Implantation in Patients with Normal Contralateral Hearing. Audiology & Neuro-Otology. 2017;21(5):286-95.
192. Doran M, Jenkinson L. Mono-syllabic word test score as a pre-operative assessment criterion for cochlear implant candidature in adults with acquired hearing loss. Cochlear Implants International: An Interdisciplinary Journal. 2016;17:13-6.
193. Shim HJ, Won JH, Moon IJ, Anderson ES, Drennan WR, McIntosh NE, et al. Can unaided non-linguistic measures predict cochlear implant candidacy? Otology & neurotology : official publication of the American Otological Society, American Neurotology Society [and] European Academy of Otology and Neurotology.35(8):1345-53.
194. Choi JE, Hong SH, Won JH, Park HS, Cho YS, Chung WH, et al. Evaluation of Cochlear Implant Candidates using a Non-linguistic Spectrotemporal Modulation Detection Test. Scientific reports.6:35235.
195. Aronoff JM, Duitsman L, Matusik DK, Hussain S, Lippmann E. Examining the Relationship Between Speech Recognition and a Spectral-Temporal Test With a Mixed Group of Hearing Aid and Cochlear Implant Users. Journal of speech, language, and hearing research : JSLHR. 2021;64(3):1073-80.
196. Drennan WR, Won JH, Timme AO, Rubinstein JT. Nonlinguistic Outcome Measures in Adult Cochlear Implant Users Over the First Year of Implantation. Ear and hearing.37(3):354-64.
197. Shrivastava MK, Eitutis ST, Lee JW, Axon PR, Donnelly NP, Tysome JR, et al. Hearing outcomes of cochlear implant recipients with pre-operatively identified cochlear dead regions. Cochlear Implants International: An Interdisciplinary Journal. 2020;21(3):160-6.
198. Tavora-Vieira D, Wedekind A, Marino R, Purdy SC, Rajan GP. Using aided cortical assessment as an objective tool to evaluate cochlear implant fitting in users with single-sided deafness. PLoS ONE [Electronic Resource]. 2018;13(2):e0193081.
199. Noble JH, Gifford RH, Hedley-Williams AJ, Dawant BM, Labadie RF. Clinical evaluation of an image-guided cochlear implant programming strategy. Audiology & Neuro-Otology. 2014;19(5):400-11.
200. Labadie RF, Noble JH, Hedley-Williams AJ, Sunderhaus LW, Dawant BM, Gifford RH. Results of Postoperative, CT-based, Electrode Deactivation on Hearing in Prelingually Deafened Adult Cochlear Implant Recipients. Otology & neurotology : official publication of the American Otological Society, American Neurotology Society [and] European Academy of Otology and Neurotology.37(2):137-45.
201. Danieli F, Dermacy T, do Amaral MSA, Reis A, Gnansia D, Hyppolito MA. Auditory performance of post-lingually deafened adult cochlear implant recipients using electrode deactivation based on postoperative cone beam CT images. European Archives of Oto Rhino Laryngology. 2020;25:25.
202. Barlow N, Purdy SC, Sharma M, Giles E, Narne V. The Effect of Short-Term Auditory Training on Speech in Noise Perception and Cortical Auditory Evoked Potentials in Adults with Cochlear Implants. Seminars in Hearing. 2016;37(1):84-98.
203. Baungaard LH, vej MG, Kroijer JS, Hestbaek MK, Samar CF, Percy-Smith L, et al. Auditory verbal skills training is a new approach in adult cochlear implant rehabilitation. Danish Medical Journal. 2019;66(3).
204. Dornhoffer JR, Reddy P, Ma C, Schvartz-Leyzac KC, Dubno JR, McRackan TR. Use of Auditory Training and Its Influence on Early Cochlear Implant Outcomes in Adults. Otology & neurotology : official publication of the American Otological Society, American Neurotology Society [and] European Academy of Otology and Neurotology. 2022;43(2):e165-e73.
205. Blom H, Marschark M, Machmer E. Simultaneous communication supports learning in noise by cochlear implant users. Cochlear Implants International: An Interdisciplinary Journal. 2017;18(1):49-56.
206. Sparrow KM, Hird K. The effectiveness of communication strategy training with adult cochlear implantees. Seminars in Hearing. 2010;31(2):165-9.
207. Oba SI, Fu QJ, Galvin 3rd JJ. Digit training in noise can improve cochlear implant users' speech understanding in noise. Ear and hearing. 2011;32(5):573-81.
208. Ihler F, Blum J, Steinmetz G, Weiss BG, Zirn S, Canis M. Development of a home-based auditory training to improve speech recognition on the telephone for patients with cochlear implants: a randomised trial. Clinical otolaryngology. 2017;42(6):1303-10.
209. Heydebr, G, Mauze E, Tye-Murray N, Binzer S, Skinner M. The efficacy of a structured group therapy intervention in improving communication and coping skills for adult cochlear implant recipients. International Journal of Audiology. 2005;44(5):272-80.

210. Schumann A, Hast A, Hoppe U. Speech performance and training effects in the cochlear implant elderly. Audiology & neuro-otology. 2014;19:45-8.
211. Schumann A, Serman M, Gefeller O, Hoppe U. Computer-based auditory phoneme discrimination training improves speech recognition in noise in experienced adult cochlear implant listeners. International journal of audiology. 2015;54(3):190-8.
212. Hutter E, Argstatter H, Grapp M, Plinkert PK. Music therapy as specific and complementary training for adults after cochlear implantation: A pilot study. Cochlear Implants International: An Interdisciplinary Journal. 2015;16:S13-S21.
213. Reis M, McMahon CM, Tavora-Vieira D, Humburg P, Boisvert I. Effectiveness of Computer-Based Auditory Training for Adult Cochlear Implant Users: A Randomized Crossover Study. Trends in hearing. 2021;25(101635698):23312165211025938.
214. Volter C, Stockmann C, Schirmer C, Dazert S. Tablet-Based Telerehabilitation Versus Conventional Face-to-Face Rehabilitation After Cochlear Implantation: Prospective Intervention Pilot Study. JMIR rehabilitation and assistive technologies. 2021;8(1):e20405.
215. Driscoll VD. The Effects of Training on Recognition of Musical Instruments by Adults with Cochlear Implants. Seminars in Hearing. 2012;33(4):410-8.
216. Reynard P, Attina V, Idriss S, Hermann R, Barilly C, Veuillet E, et al. Effect of Serious Gaming on Speech-in-Noise Intelligibility in Adult Cochlear Implantees: A Randomized Controlled Study. Journal of clinical medicine. 2022;11(10).
217. Anderson CA, Wiggins IM, Kitterick PT, Hartley DEH. Pre-operative Brain Imaging Using Functional Near-Infrared Spectroscopy Helps Predict Cochlear Implant Outcome in Deaf Adults. JARO - Journal of the Association for Research in Otolaryngology. 2019;20(5):511-28.
218. Ichikawa K, Kashio A, Mori H, Ochi A, Karino S, Sakamoto T, et al. A new computed tomography method to identify meningitis-related cochlear ossification and fibrosis before cochlear implantation. Otolaryngology-Head & Neck Surgery. 2014;150(4):646-53.
219. Arnoldner C, Schwarz-Nemec U, Auinger AB, Yildiz E, Matula C, Dahm V. A novel scoring system based on small vestibular schwannomas to determine consideration for cochlear implantation. Clinical otolaryngology : official journal of ENT-UK ; official journal of Netherlands Society for Oto-Rhino-Laryngology & Cervico-Facial Surgery. 2021;46(6):1223-8.
220. Rees J, Abrar R, Stapleton E. A comparison of imaging techniques to measure skin flap thickness in cochlear implant patients to enable pre-operative device selection. Cochlear implants international. 2022;23(4):179-88.
221. Daoudi H, Lahlou G, Torres R, Sterkers O, Lefeuvre V, Ferrary E, et al. Robot-assisted Cochlear Implant Electrode Array Insertion in Adults: A Comparative Study With Manual Insertion. Otology & Neurotology. 2020;10:10.
222. Rupp R, Hornung J, Balk M, Hoppe U, Iro H, Gostian A-O. Ultrasound in Diagnosis of Magnet Dislocation of Cochlear Implants: A Retrospective Study in Patients With Surgical Magnet Repositioning and Preinterventional Ultrasound Examination. Otology & Neurotology. 2020;41(9):e1098-e104.
223. Nafaji S, Abshirini H, Karkhaneh S. Comparison of vestibular rehabilitation on balance function in cochlear implant recipients. The international tinnitus journal. 2021;25(1):10-2.
224. Howe S, Mawman D. Audit of adult post-implant annual reviews and evaluation of patient-led review. Cochlear Implants International: An Interdisciplinary Journal. 2015;16(1):3-8.
225. Garber S, Ridgely MS, Bradley M, Chin KW. Payment under public and private insurance and access to cochlear implants. Archives of Otolaryngology -- Head & Neck Surgery.128(10):1145-52.
226. Sorkin DL. Impact of Medicaid on Cochlear Implant Access. Otology & neurotology : official publication of the American Otological Society, American Neurotology Society [and] European Academy of Otology and Neurotology.40(3):e336-e41.
227. Bu DD, Schwam ZG, Neifert SN, Ferrandino R, Kaul VZ, Perez E, et al. The Price of Otologic Procedures: Variation in Markup by Surgical Procedure and Geography in the United States. Otology & neurotology : official publication of the American Otological Society, American Neurotology Society [and] European Academy of Otology and Neurotology. 2021;42(8):1184-91.
228. Looi V, Bluett C, Boisvert I. Referral rates of postlingually deafened adult hearing aid users for a cochlear implant candidacy assessment. International Journal of Audiology. 2017;56(12):919-25.
229. Raine C, Atkinson H, Strachan DR, Martin JM. Access to cochlear implants: Time to reflect. Cochlear Implants International: An Interdisciplinary Journal. 2016;17:42-6.
230. Mistry D, Ryan J, Maessen H, Bance M. Differences in perception of hearing handicap between cochlear implant users and their spouses. Laryngoscope.124(5):1199-203.
231. Sydlowski S, Donovan C, McNair J, Hahn K, Petter K, Marks T, et al. Optimizing Care Delivery by Applying an Innovative Shared Medical Appointment Model for Determination of Cochlear Implant Candidacy. Otology & neurotology : official publication of the American Otological Society, American Neurotology Society [and] European Academy of Otology and Neurotology. 2021;42(8):e1008-e12.
232. Chen SY, Grisel JJ, Lam A, Golub JS. Assessing Cochlear Implant Outcomes in Older Adults Using HERMES: A National Web-based Database. Otology & neurotology : official publication of the American Otological Society, American Neurotology Society [and] European Academy of Otology and Neurotology. 2017;38(10):e405-e12.

233. Causon A, Verschuur C, Newman TA. Trends in cochlear implant complications: implications for improving long-term outcomes. Otology & neurotology : official publication of the American Otological Society, American Neurotology Society [and] European Academy of Otology and Neurotology. 2013;34(2):259-65.
234. Bestourous DE, Davidson L, Reilly BK. A Review of Reported Adverse Events in MRI-Safe and MRI-Conditional Cochlear Implants. Otology & neurotology : official publication of the American Otological Society, American Neurotology Society [and] European Academy of Otology and Neurotology. 2022;43(1):42-7.
235. Grisel J, Miller S, Schafer EC. A Novel Performance-Based Paradigm of Care for Cochlear Implant Follow-Up. The Laryngoscope. 2022;132(8607378):S1-S10.
236. Tognola G, Murri A, Cuda D. An Application of eHealth Technology Toward the Digitization of the Health Records of Older Patients With Cochlear Implants. American journal of audiology. 2019(3):796-801.
237. Smith JG, Metzger NL. Evaluation of pneumococcal vaccination rates after vaccine protocol changes and nurse education in a tertiary care teaching hospital. Journal of Managed Care Pharmacy.17(9):701-8.
238. Miller SE, Anderson C, Manning J, Schafer E. Insurance Payer Status Predicts Postoperative Speech Outcomes in Adult Cochlear Implant Recipients. Journal of the American Academy of Audiology.31(9):666-73.
239. Dunn C, Miller SE, Schafer EC, Silva C, Gifford RH, Grisel JJ. Benefits of a Hearing Registry: Cochlear Implant Candidacy in Quiet Versus Noise in 1,611 Patients. American Journal of Audiology. 2020;29(4):851-61.
240. Ngombu SJ, Ray C, Vasil K, Moberly AC, Varadarajan VV. Development of a novel screening tool for predicting Cochlear implant candidacy. Laryngoscope investigative otolaryngology. 2021;6(6):1406-13.
241. Reddy P, Dornhoffer JR, Camposeo EL, Dubno JR, McRackan TR. Using Clinical Audiologic Measures to Determine Cochlear Implant Candidacy. Audiology & neuro-otology. 2022;27(3):235-42.
242. So RJ, Padova D, Bowditch S, Agrawal Y. Candidacy for Cochlear implantation: Validating a novel Cochlear implant candidacy calculator against gold-standard, in-clinic audiometric assessments. Laryngoscope investigative otolaryngology. 2022;7(3):835-9.
243. Copson B, Wijewickrema S, Zhou Y, Piromchai P, Briggs R, Bailey J, et al. Supporting skill acquisition in cochlear implant surgery through virtual reality simulation. Cochlear implants international.18(2):89-96.
244. Frendo M, Frithioff A, Konge L, Sorensen MS, Andersen SAW. Cochlear implant surgery: Learning curve in virtual reality simulation training and transfer of skills to a 3D-printed temporal bone - A prospective trial. Cochlear implants international. 2021;22(6):330-7.
245. Canfarotta MW, Dillon MT, Buss E, Pillsbury HC, Brown KD, O'Connell BP. Validating a New Tablet-based Tool in the Determination of Cochlear Implant Angular Insertion Depth. Otology & neurotology : official publication of the American Otological Society, American Neurotology Society [and] European Academy of Otology and Neurotology.40(8):1006-10.
246. D'Haese PSC, Van Rompaey V, De Bodt M, Van de Heyning P. Can a Digital Awareness Campaign Change Knowledge and Beliefs Regarding Cochlear Implants? A Study in Older Adults in 5 European Countries. Inquiry (00469580). 2020;57:1-6.
247. Hoppe U, Hast A, Hocke T. Audiometry-Based Screening Procedure for Cochlear Implant Candidacy. Otology & neurotology : official publication of the American Otological Society, American Neurotology Society [and] European Academy of Otology and Neurotology. 2015;36(6):1001-5.
248. Gubbels SP, Gartrell BC, Ploch JL, Hanson KD. Can routine office-based audiometry predict cochlear implant evaluation results? Laryngoscope. 2017;127(1):216-22.
249. Ramos-Miguel A, Perez-Zaballos T, Perez D, Falconb JC, Ramosb A. Use of data mining to predict significant factors and benefits of bilateral cochlear implantation. European Archives of Oto-Rhino-Laryngology.272(11):3157-62.
250. McRackan TR, Noble JH, Wilkinson EP, Mills D, Dietrich MS, Dawant BM, et al. Implementation of Image-Guided Cochlear Implant Programming at a Distant Site. Otolaryngology-Head & Neck Surgery. 2017;156(5):933-7.
251. Meeuws M, Pascoal D, Bermejo I, Artaso M, De Ceulaer G, Govaerts PJ. Computer-assisted CI fitting: Is the learning capacity of the intelligent agent FOX beneficial for speech understanding? Cochlear Implants International: An Interdisciplinary Journal. 2017;18(4):198-206.
252. Battmer RD, Borel S, Brendel M, Buchner A, Cooper H, Fielden C, et al. Assessment of 'Fitting to Outcomes Expert' FOX™ with new cochlear implant users in a multi-centre study. Cochlear implants international. 2015;16(2):100-9.
253. Buechner A, Vaerenberg B, Gazibegovic D, Brendel M, De Ceulaer G, Govaerts P, et al. Evaluation of the 'Fitting to Outcomes eXpert' (FOX) with established cochlear implant users. Cochlear implants international. 2015;16(1):39-46.
254. Crowson MG, Dixon P, Mahmood R, Lee JW, Shipp D, Le T, et al. Predicting Postoperative Cochlear Implant Performance Using Supervised Machine Learning. Otology & Neurotology. 2020;41(8):e1013-e23.
255. Rouf CE, Lescanne E, Villeneuve A, Reffet K, Kim S, Bakhos D. Impact of a multimedia support on the understanding of medical information by hearing-impaired patients before cochlear implantation. European Annals of Otorhinolaryngology, Head and Neck Diseases.134(6):387-92.
256. Seymour N, Lakhani R, Hartley B, Cochrane L, Jephson C. Cochlear implantation: An assessment of quality and readability of web-based information aimed at patients. Cochlear Implants International: An Interdisciplinary Journal. 2015;16(6):321-5.
257. Stacey PC, Raine CH, O'Donoghue GM, Tapper L, Twomey T, Summerfield AQ. Effectiveness of computer-based auditory training for adult users of cochlear implants. International Journal of Audiology. 2010;49(5):347-56.
258. Smith L, Bartel L, Joglekar S, Chen J. Musical Rehabilitation in Adult Cochlear Implant Recipients With a Self-administered Software. Otology & Neurotology. 2017;38(8):e262-e7.

259. Wolfe J, Morais Duke M, Schafer E, Cire G, Menapace C, O'Neill L. Evaluation of a wireless audio streaming accessory to improve mobile telephone performance of cochlear implant users. International Journal of Audiology. 2016;55(2):75-82.
260. Wolfe J, Morais M, Schafer E. Speech Recognition of Bimodal Cochlear Implant Recipients Using a Wireless Audio Streaming Accessory for the Telephone. Otology & neurotology : official publication of the American Otological Society, American Neurotology Society [and] European Academy of Otology and Neurotology.37(2):e20-e5.
261. Nunez-Batalla F, Fern, ez-Junquera AB, Suarez-Villanueva L, Diaz-Fresno E, oval-Menendez I, et al. Application of Wireless Contralateral Routing of Signal (CROS) Technology in Unilateral Cochlear Implant Users. Acta Otorrinolaringologica Espanola. 2020.
262. Snapp HA, Hoffer ME, Spahr A, Rajguru S. Application of Wireless Contralateral Routing of Signal Technology in Unilateral Cochlear Implant Users with Bilateral Profound Hearing Loss. Journal of the American Academy of Audiology. 2019;30(7):579-89.
263. Arora R, Amoodi H, Stewart S, Friesen L, Lin V, Nedzelski J, et al. The addition of a contralateral routing of signals microphone to a unilateral cochlear implant system--a prospective study in speech outcomes. Laryngoscope.123(3):746-51.
264. Dwyer RT, Kessler D, Gifford RH, Butera IM. Contralateral Routing of Signal Yields Significant Speech in Noise Benefit for Unilateral Cochlear Implant Recipients. Journal of the American Academy of Audiology. 2019;30(3):235-44.
265. Grewal AS, Kuthubutheen J, Smilsky K, Nedzelski JM, Chen JM, Friesen L, et al. The role of a new contralateral routing of signal microphone in established unilateral cochlear implant recipients. Laryngoscope.125(1):197-202.
266. Gawliczek T, Guignard J, Schmid C, Wimmer W, Caversaccio M, Kompis M, et al. Using a cochlear implant processor as contralateral routing of signals device in unilateral cochlear implant recipients. European archives of oto-rhino-laryngology : official journal of the European Federation of Oto-Rhino-Laryngological Societies (EUFOS) : affiliated with the German Society for Oto-Rhino-Laryngology - Head and Neck Surgery. 2022;279(2):645-52.
267. Hoshino ACH, Goffi-Gomez MVS, Sierra PAS, Agrawal S, Rodriguez C, Carvalho ACMd, et al. Can the use of the CROS system provide head shadow effect contribution to unilateral Cochlear Implant Users? CoDAS. 2022;34(5):e20210071.
268. van Loon MC, Goverts ST, Merkus P, Hensen EF, Smits C. The addition of a contralateral microphone for unilateral cochlear implant users: not an alternative for bilateral cochlear implantation. Otology & neurotology : official publication of the American Otological Society, American Neurotology Society [and] European Academy of Otology and Neurotology.35(9):e233-e9.
269. Wimmer W, Kompis M, Stieger C, Caversaccio M, Weder S. Directional Microphone Contralateral Routing of Signals in Cochlear Implant Users: A Within-Subjects Comparison. Ear Hear. 2017;38(3):368-73.
270. Vroegop JL, Dingemanse JG, Homans NC, Goedegebure A. Evaluation of a wireless remote microphone in bimodal cochlear implant recipients. International Journal of Audiology. 2017;56(9):643-9.
271. Wesarg T, Arndt S, Wiebe K, Schmid F, Huber A, Mülder HE, et al. Speech Recognition in Noise in Single-Sided Deaf Cochlear Implant Recipients Using Digital Remote Wireless Microphone Technology. Journal of the American Academy of Audiology. 2019;30(7):607-18.
272. Wesarg T, Stelzig Y, Hilgert-Becker D, Kathage B, Wiebe K, Aschendorff A, et al. Application of Digital Remote Wireless Microphone Technology in Single-Sided Deaf Cochlear Implant Recipients. Journal of the American Academy of Audiology.31(4):246-56.
273. Duke MM, Wolfe J, Schafer E. Recognition of speech from the television with use of a wireless technology designed for cochlear implants. Journal of the American Academy of Audiology.27(5):388-94.
274. Tyler RS, Owen RL, Bridges J, er PE, Perreau A, Mancini PC. Tinnitus Suppression in Cochlear Implant Patients Using a Sound Therapy App. American Journal of Audiology. 2018;27(3):316-23.
275. Perreau AE, Tyler RS, Frank V, Watts A, Mancini PC. Use of a Smartphone App for Cochlear Implant Patients With Tinnitus. American journal of audiology. 2021;30(3):676-87.
276. Philips B, Smits C, Govaerts PJ, Doorn I, Vanpoucke F. Empowering Senior Cochlear Implant Users at Home via a Tablet Computer Application. American Journal of Audiology. 2018;27:417-30.
277. Schepers K, Steinhoff H-J, Ebenhoch H, Böck K, Bauer K, Rupprecht L, et al. Remote programming of cochlear implants in users of all ages. Acta Oto-Laryngologica. 2019;139(3):251-7.
278. Luryi AL, Tower JI, Preston J, Burkl, A, Trueheart CE, et al. Cochlear Implant Mapping Through Telemedicine-A Feasibility Study. Otology & neurotology : official publication of the American Otological Society, American Neurotology Society [and] European Academy of Otology and Neurotology.41(3):e330-e3.
279. Samuel PA, Goffi-Gomez MV, Bittencourt AG, Tsuji RK, Brito R. Remote programming of cochlear implants. Codas.26(6):481-6.
280. Shapiro SB, Lipschitz N, Kemper N, Zuccarello M, Breen JT, Pensak ML, et al. Early Experience With Telemedicine in Patients Undergoing Otologic/Neurotologic Procedures. Otology & Neurotology. 2020;41(9):e1154-e7.
281. Browning LM, Nie Y, Rout A, Heiner M. Audiologists' preferences in programming cochlear implants: A preliminary report. Cochlear implants international.1-13.
282. Fielden CA, Kitterick PT. Contralateral acoustic hearing aid use in adult unilateral cochlear implant recipients: Current provision, practice, and clinical experience in the UK. Cochlear Implants International: An Interdisciplinary Journal. 2016;17(3):132-45.

283. Scherf FW, Arnold LP, Poster presentation at the 12th International Conference on Cochlear I, Other Implantable Auditory Technologies EAtN, France sSP. Exploring the clinical approach to the bimodal fitting of hearing aids and cochlear implants: results of an international survey. Acta Oto-Laryngologica.134(11):1151-7.
284. Vaerenberg B, Smits C, De Ceulaer G, Zir E, Harman S, Jaspers N, et al. Cochlear implant programming: a global survey on the state of the art. The scientific world journal. 2014;2014:501738.
285. Carlson ML, O'Connell BP, Lohse CM, Driscoll CL, Sweeney AD. Survey of the American Neurotology Society on Cochlear Implantation: Part 2, Surgical and Device-Related Practice Patterns. Otology & neurotology : official publication of the American Otological Society, American Neurotology Society [and] European Academy of Otology and Neurotology.39(1):e20-e7.
286. Carlson ML, Sladen DP, Gurgel RK, Tombers NM, Lohse CM, Driscoll CL. Survey of the American Neurotology Society on Cochlear Implantation: Part 1, Candidacy Assessment and Expanding Indications. Otology & neurotology : official publication of the American Otological Society, American Neurotology Society [and] European Academy of Otology and Neurotology.39(1):e12-e9.
287. D'Haese PSC, Van Rompaey V, De Bodt M, Van de Heyning P. The knowledge and beliefs regarding practical aspects of cochlear implants: A study of otorhinolaryngologists in a secondary setting in a multi-country study. Cochlear Implants International: An Interdisciplinary Journal. 2018;19(1):14-21.
288. Manrique M, Ramos A, Pradel B, Cenjor C, Calavia D, Morera C. Survey on the knowledge of cochlear implant indication in the treatment of hearing loss in Spain. Acta Otorrinolaringologica Espanola.69(5):251-9.
289. Reddy YM, Anjali L, Augustine A, Mary J, Ajay P. Knowledge, attitude and practices regarding cochlear implants among medical doctors other than otolaryngologists: a prospective cross-sectional study. The Journal of laryngology and otology. 2022;136(6):492-9.
290. Saunders J, Barrs D. Cochlear implantation in developing countries as humanitarian service: physician attitudes and recommendations for best practice. Otolaryngology - Head & Neck Surgery.145(1):74-9.
291. Chundu S, Buhagiar R. Audiologists' knowledge of cochlear implants and their related referrals to the cochlear implant centre: Pilot study findings from UK. Cochlear Implants International: An Interdisciplinary Journal. 2013;14(4):213-24.
292. Prentiss S, ra, Snapp H, Zwolan T. Audiology Practices in the Preoperative Evaluation and Management of Adult Cochlear Implant Candidates. JAMA Otolaryngology-Head & Neck Surgery. 2020;146(2):136-42.
293. Peters BR, Wyss J, Manrique M. Worldwide trends in bilateral cochlear implantation. Laryngoscope.120:S17-S44.
294. Schwartz SR, Watson SD, Backous DD. Assessing candidacy for bilateral cochlear implants: A survey of practices in the United States and Canada. Cochlear Implants International: An Interdisciplinary Journal. 2012;13(2):86-92.
295. Vickers D, De Raeve L, Graham J. International survey of cochlear implant candidacy. Cochlear Implants International: An Interdisciplinary Journal. 2016;17:36-41.
296. Cohen SM, Labadie RF, Haynes DS. Primary care approach to hearing loss: the hidden disability. Ear, Nose, & Throat Journal. 2005;84(1):26, 9-31, 44.
297. Olson AD, Pugh BB, Bishop M. Knowledge about Cochlear Implants among Vocational Rehabilitation Counselors. Journal of Applied Rehabilitation Counseling.40(1):27-34.
298. Rossi-Katz J, Arehart KH. Survey of Audiologic Service Provision to Older Adults With Cochlear Implants. American Journal of Audiology. 2011;20(2):84-9.
299. Constable JD, Broomfield SJ, Romeo E, Clamp PJ. The potential effect of the updated national criteria on adult cochlear implantation in England and Wales. Cochlear implants international. 2022;23(3):119-24.
300. Group UCIS. Criteria of Candidacy for Unilateral Cochlear Implantation in Postlingually Deafened Adults II: Cost-Effectiveness Analysis. Ear and Hearing. 2004;25(4):336-60.
301. Cutler H, Gumbie M, Olin E, Parkinson B, Bowman R, Quadri H, et al. The cost-effectiveness of unilateral cochlear implants in UK adults. The European journal of health economics : HEPAC : health economics in prevention and care. 2022;23(5):763-79.
302. Gumbie M, Olin E, Parkinson B, Bowman R, Cutler H. The cost-effectiveness of Cochlear implants in Swedish adults. BMC health services research. 2021;21(1):319.
303. Dreyfuss M, Giat Y, Veraguth D, Roosli C, Huber AM, Laske RD. Cost Effectiveness of Cochlear Implantation in Single-Sided Deafness. Otology & neurotology : official publication of the American Otological Society, American Neurotology Society [and] European Academy of Otology and Neurotology. 2021;42(8):1129-35.
304. Laske RD, Dreyfuss M, Stulman A, Veraguth D, Huber A, M. e, et al. Age Dependent Cost-Effectiveness of Cochlear Implantation in Adults. Is There an Age Related Cut-off? Otology & Neurotology. 2019;40(7):892-9.
305. Molinier L, Bocquet H, Bongard V, Fraysse B. The economics of cochlear implant management in France: A multicentre analysis. European Journal of Health Economics.10(3):347-55.
306. Trinidade A, Page JC, Kennett SW, Cox MD, Dornhoffer JL. Simultaneous versus sequential bilateral cochlear implants in adults: Cost analysis in a US setting. Laryngoscope. 2017;127(11):2615-8.
307. Thum C, Lenarz T, Lesinski-Schiedat A, Flessa S. Lifetime cost of unilateral cochlear implants in adults: a Monte Carlo simulation. European Journal of Health Economics. 2020.
308. Brown CS, Choi KJ, Kaylie DM. Preoperative Imaging Findings and Cost in Adults With Postlingual Deafness Prior to Cochlear Implant. Annals of Otology, Rhinology and Laryngology.127(4):270-4.

309. Reis M, Boisvert I, Beedell E, Mumford V. Auditory Training for Adult Cochlear Implant Users: A Survey and Cost Analysis Study. Ear and hearing. 2019;40(6):1445-56.
310. Summerfield AQ, Marshall DH, Barton GR, Bloor KE. A cost-utility scenario analysis of bilateral cochlear implantation. Archives of Otolaryngology -- Head & Neck Surgery.128(11):1255-62.
311. Skarzynski PH, Ciesla K, Lorens A, Wojcik J, Skarzynski H. Cost-Utility Analysis of Bilateral Cochlear Implantation in Adults With Severe to Profound Sensorineural Hearing Loss in Poland. Otology & neurotology : official publication of the American Otological Society, American Neurotology Society [and] European Academy of Otology and Neurotology. 2021;42(5):706-12.
312. Chen JM, Amoodi H, Mittmann N. Cost-utility analysis of bilateral cochlear implantation in adults: A health economic assessment from the perspective of a publicly funded program. Laryngoscope. 2014;124(6):1452-8.
313. Smulders YE, van Zon A, Stegeman I, van Zanten GA, Rinia AB, Stokroos RJ, et al. Cost-Utility of Bilateral Versus Unilateral Cochlear Implantation in Adults: a Randomized Controlled Trial. Otology & neurotology. 2016;37(1):38-45.
314. Lee H, Park E, Kim HJ, Choi J, Kim H. Cost-utility analysis of cochlear implants in Korea using different measures of utility. Acta Oto-Laryngologica. 2006;126(8):817-23.
315. Seebacher J, Muigg F, Kuhn H, Weichbold V, Galvan O, Zorowka P, et al. Cost-utility Analysis of Cochlear Implantation in Adults With Single-sided Deafness: Austrian and German Perspective. Otology & neurotology : official publication of the American Otological Society, American Neurotology Society [and] European Academy of Otology and Neurotology. 2021;42(6):799-805.
316. Clinkard D, Barbic S, Amoodi H, Shipp D, Lin V. The economic and societal benefits of adult cochlear implant implantation: A pilot exploratory study. Cochlear Implants Int. 2015;16(4):181-5.
317. Monteiro E, Shipp D, Chen J, Nedzelski J, Lin V. Cochlear implantation: a personal and societal economic perspective examining the effects of cochlear implantation on personal income. Journal of Otolaryngology: Head and Neck Surgery.41:S43-8.
318. Neve OM, Boerman JA, van den Hout WB, Briaire JJ, van Benthem PPG, Frijns JHM. Cost-benefit Analysis of Cochlear Implants: A Societal Perspective. Ear and hearing. 2021;42(5):1338-50.
319. Kos MI, Degive C, Boex C, Guyot JP. Professional occupation after cochlear implantation. Journal of Laryngology and Otology.121(3):215-8.
320. Claeson M, Ringdahl A. Prevalence of cochlear implants in postlingually deafened adults in a Swedish region. International Journal of Audiology. 2006;45(11):670-4.
321. De Raeve L, Archbold S, Lehnhardt-Goriany M, Kemp T. Prevalence of cochlear implants in Europe: trend between 2010 and 2016. Cochlear Implants International.21(5):275-80.
322. De Raeve L, van Hardeveld R. PREVALENCE OF COCHLEAR IMPLANTS IN EUROPE: WHAT DO WE KNOW AND WHAT CAN WE EXPECT? Journal of Hearing Science. 2013;3(4):9-16.
323. Saito K, Fujita T, Osaki Y, Koyama H, Shiraishi K, Kobayashi T, et al. Prevalence of potential candidates for electric-acoustic stimulation implant in a hearing-impaired population. Auris Nasus Larynx. 2019.
324. Agabigum B, Mir A, Arianpour K, Svider PF, Walsh EM, Hong RS. Evolving Trends in Cochlear Implantation: A Critical Look at the Older Population. Otology & neurotology : official publication of the American Otological Society, American Neurotology Society [and] European Academy of Otology and Neurotology. 2018;39(8):e660-e4.
325. Mahboubi H, Lin HW, Bhattacharyya N. Prevalence, Characteristics, and Treatment Patterns of Hearing Difficulty in the United States. JAMA Otolaryngology-Head & Neck Surgery. 2018;144(1):65-70.
326. Aldhafeeri AM, Alzhrani F, Alajlan S, AlSanosi A, Hagr A. Clinical profile and management of revision cochlear implant surgeries. Saudi medical journal. 2021;42(2):223-7.
327. Nassiri AM, Sorkin DL, Carlson ML. Current Estimates of Cochlear Implant Utilization in the United States. Otology & neurotology : official publication of the American Otological Society, American Neurotology Society [and] European Academy of Otology and Neurotology. 2022;43(5):e558-e62.
328. Holder JT, Reynolds SM, Sunderhaus LW, Gifford RH. Current Profile of Adults Presenting for Preoperative Cochlear Implant Evaluation. Trends in hearing.22:2331216518755288.
329. Barnes JH, Yin LX, Marinelli JP, Carlson ML. Audiometric Profile of Cochlear Implant Recipients Demonstrates Need for Revising Insurance Coverage. Laryngoscope. 2020;21:21.
330. Fitzpatrick EM, Brewster L. Adult Cochlear Implantation in Canada: Results of a Survey. Canadian Journal of Speech-Language Pathology & Audiology. 2010;34(4):290-6.
331. Hixon B, Chan S, Adkins M, Shinn JB, Bush ML. Timing and Impact of Hearing Healthcare in Adult Cochlear Implant Recipients: A Rural-Urban Comparison. Otology & neurotology : official publication of the American Otological Society, American Neurotology Society [and] European Academy of Otology and Neurotology.37(9):1320-4.
332. Balachandra S, Xierali IM, Nivet MA, Hunter JB. Trends in Cochlear Implantation in Texas: An Exploration of Outpatient Discharge Data, 2010 to 2017. The Annals of otology, rhinology, and laryngology. 2022;131(1):86-93.
333. Kay-Rivest E, Irace AL, Golub JS, Svirsky MA. Prevalence of Single-Sided Deafness in the United States. The Laryngoscope. 2022;132(8):1652-6.
334. Yamaguchi CT, Goffi-Gomez MV. Prevalence of contralateral hearing aid use in adults with cochlear implants. International @rchives of Otorhinolaryngology.17(4):370-4.

335. Devocht EM, George EL, Janssen AM, Stokroos RJ. Bimodal Hearing Aid Retention after Unilateral Cochlear Implantation. Audiology & Neuro-Otology. 2015;20(6):383-93.
336. Tolisano AM, Fang LB, Kutz JW, Isaacson B, Hunter JB. Better defining best-aided condition: The role of hearing aids on cochlear implantation qualification rates. American Journal of Otolaryngology - Head and Neck Medicine and Surgery. 2020.
337. Bhatt YM, Green KMJ, Mawman DJ, Aplin Y, O'Driscoll MP, Saeed SR, et al. Device nonuse among adult cochlear implant recipients. Otology and Neurotology.26(2):183-7.
338. Távora-Vieira D, Acharya A, Rajan GP. What can we learn from adult cochlear implant recipients with single-sided deafness who became elective non-users? Cochlear Implants International: An Interdisciplinary Journal. 2020;21(4):220-7.
339. Imagawa N, Hirota E, Morino T, Kojima H, Kojima H. Factors related to the satisfaction level of elderly hearing-impaired individuals with cochlear implants. Auris Nasus Larynx. 2020;47(5):793-9.
340. Holder JT, Mayberry LS, Gifford R. The Cochlear Implant Use Questionnaire: Assessing Habits and Barriers to Use. Otology & neurotology : official publication of the American Otological Society, American Neurotology Society [and] European Academy of Otology and Neurotology. 2022;43(1):e23-e9.
341. Tolisano AM, Schauwecker N, Baumgart B, Whitson J, Kutz JW, Isaacson B, et al. Identifying Disadvantaged Groups for Cochlear Implantation: Demographics from a Large Cochlear Implant Program. Annals of Otology, Rhinology and Laryngology.129(4):347-54.
342. Dornhoffer JR, Holcomb MA, Meyer TA, Dubno JR, McRackan TR. Factors Influencing Time to Cochlear Implantation. Otology & Neurotology.41(2):173-7.
343. Turunen-Taheri SK, Edén M, Hellström S, Carlsson P-I. Rehabilitation of adult patients with severe-to-profound hearing impairment – why not cochlear implants? Acta Oto-Laryngologica. 2019;139(7):604-11.
344. Henkin Y, Shapira Y, Yaar Soffer Y. Current demographic and auditory profiles of adult cochlear implant candidates and factors affecting uptake. International journal of audiology. 2022;61(6):483-9.
345. Redmann AJ, Tawfik K, Hammer T, Wenstrup L, Stevens S, Breen JT, et al. Determining treatment choices after the cochlear implant evaluation process. Laryngoscope investigative otolaryngology. 2021;6(2):320-4.
346. Thompson L, Bazeer HZ, Young B, Smith G, Blackaby J, Wasson J, et al. Cochlear implant eligibility in an adult hearing aid population: a multi-perspective service evaluation of a patient referral pathway at a British district general hospital. The Journal of laryngology and otology. 2022;136(8):755-9.
347. Balachandra S, Tolisano AM, Qazi S, Hunter JB. Self-Identified Patient Barriers to Pursuit of Cochlear Implantation. Otology & neurotology : official publication of the American Otological Society, American Neurotology Society [and] European Academy of Otology and Neurotology. 2021;42(10):S26-S32.
348. Sucher CM, Eikelboom RH, Stegeman I, Jayakody DMP, Atlas MD. The effect of hearing loss configuration on cochlear implantation uptake rates: an Australian experience. International Journal of Audiology.59(11):828-34.
349. Mahendran GN, Rosenbluth T, Featherstone M, Vivas EX, Mattox DE, Hobson CE. Racial Disparities in Adult Cochlear Implantation. Otolaryngology--head and neck surgery : official journal of American Academy of Otolaryngology-Head and Neck Surgery. 2022;166(6):1099-105.
350. Nassiri AM, Holcomb MA, Perkins EL, Bucker AL, Prentiss SM, Welch CM, et al. Catchment Profile of Large Cochlear Implant Centers in the United States. Otolaryngology--head and neck surgery : official journal of American Academy of Otolaryngology-Head and Neck Surgery. 2022;167(3):545-51.
351. Raymond MJ, Dong A, Naissir SB, Vivas EX. Postoperative Healthcare Utilization of Elderly Adults After Cochlear Implantation. Otology & neurotology : official publication of the American Otological Society, American Neurotology Society [and] European Academy of Otology and Neurotology.41(2):208-13.
352. Spitzer JB, Cellum IP, Bosworth C. Stability of audiometric measures and challenges in long-term management of the elderly cochlear implant patient. Otology & neurotology : official publication of the American Otological Society, American Neurotology Society [and] European Academy of Otology and Neurotology.34(9):1636-41.
353. D'Haese PSC, De Bodt M, Van Rompaey V, Van de Heyning P. Awareness of Hearing Loss in Older Adults: Results of a Survey Conducted in 500 Subjects Across 5 European Countries as a Basis for an Online Awareness Campaign. Inquiry : a journal of medical care organization, provision and financing. 2018;55:46958018759421.
354. Smit JV, Pielkenrood BJ, Arts RAGJ, Janssen M, L. a, Temel Y, et al. Patient Acceptance of Invasive Treatments for Tinnitus. American Journal of Audiology. 2018;27(2):184-96.
355. Dewey RS, Kitterick PT. Cochlear implant user perceptions of magnetic resonance imaging. Cochlear implants international. 2022;23(1):11-20.
356. Zhang L, Ding AS, Xie DX, Creighton FX. Understanding Public Perceptions Regarding Cochlear Implant Surgery in Adults. Otology & neurotology : official publication of the American Otological Society, American Neurotology Society [and] European Academy of Otology and Neurotology. 2022;43(3):e331-e6.
357. Aylward A, Gordon SA, Murphy-Meyers M, Allen CM, Patel NS, Gurgel RK. Caregiver Quality of Life After Cochlear Implantation in Older Adults. Otology & neurotology : official publication of the American Otological Society, American Neurotology Society [and] European Academy of Otology and Neurotology. 2022;43(2):e191-e7.
